# TGFβ-Signaling and FOXG1-Expression Are a Hallmark of Astrocyte Lineage Diversity in the Murine Ventral and Dorsal Forebrain

**DOI:** 10.3389/fncel.2018.00448

**Published:** 2018-11-28

**Authors:** Stefan Christopher Weise, Alejandro Villarreal, Stefanie Heidrich, Fariba Dehghanian, Christian Schachtrup, Sigrun Nestel, Jennifer Schwarz, Kathrin Thedieck, Tanja Vogel

**Affiliations:** ^1^Department of Molecular Embryology, Institute of Anatomy and Cell Biology, Medical Faculty, University of Freiburg, Freiburg, Germany; ^2^Faculty of Biology, University of Freiburg, Freiburg, Germany; ^3^Department of Neuroanatomy, Institute of Anatomy and Cell Biology, Medical Faculty, University of Freiburg, Freiburg, Germany; ^4^Division of Genetics, Department of Biology, Faculty of Sciences, University of Isfahan, Isfahan, Iran; ^5^Department of Biochemistry and Functional Proteomics, Faculty of Biology, University of Freiburg, Freiburg, Germany; ^6^Spemann Graduate School of Biology and Medicine, University of Freiburg, Freiburg, Germany; ^7^Section of Systems Medicine of Metabolism and Signaling, Department of Pediatrics and University Medical Center Groningen, University of Groningen, Groningen, Netherlands; ^8^Department of Neuroscience, School of Medicine and Health Sciences, Carl von Ossietzky University of Oldenburg, Oldenburg, Germany

**Keywords:** lineage-tracing, neural differentiation, SILAC, Tgfbr2 knockout, astrocyte-diversity

## Abstract

Heterogeneous astrocyte populations are defined by diversity in cellular environment, progenitor identity or function. Yet, little is known about the extent of the heterogeneity and how this diversity is acquired during development. To investigate the impact of TGF (transforming growth factor) β-signaling on astrocyte development in the telencephalon we deleted the TGFBR2 (transforming growth factor beta receptor 2) in early neural progenitor cells in mice using a FOXG1 (forkhead box G1)-driven CRE-recombinase. We used quantitative proteomics to characterize TGFBR2-deficient cells derived from the mouse telencephalon and identified differential protein expression of the astrocyte proteins GFAP (glial fibrillary acidic protein) and MFGE8 (milk fat globule-EGF factor 8). Biochemical and histological investigations revealed distinct populations of astrocytes in the dorsal and ventral telencephalon marked by GFAP or MFGE8 protein expression. The two subtypes differed in their response to TGFβ-signaling. Impaired TGFβ-signaling affected numbers of GFAP astrocytes in the ventral telencephalon. In contrast, TGFβ reduced MFGE8-expression in astrocytes deriving from both regions. Additionally, lineage tracing revealed that both GFAP and MFGE8 astrocyte subtypes derived partly from FOXG1-expressing neural precursor cells.

## Introduction

The development of the vertebrate forebrain relies on a timely regulated specification of different neural cell types. During this process, symmetric and asymmetric divisions of radial glia cells (RGCs) lead to the generation of neurons, astrocytes and oligodendrocytes. Differentiation of these cell types is temporally regulated whereby neurogenesis precedes astrogliogenesis and oligodendrocyte formation (Sauvageot and Stiles, 2002; Miller and Gauthier, 2007; Pinto and Götz, 2007; Franco and Müller, 2013). Transcriptional programs that favor specific differentiation programs are controlled intrinsically and extrinsically through activation of diverse signaling pathways (Miller and Gauthier, 2007). TGFβ ligands (TGFβ1, 2, and 3) are among extrinsic signaling molecules with the competence to initiate specific differentiation programs of neural stem cells in different allocations.

In the spinal cord, mid- and hindbrain, TGFβ controls temporal generation of neural and glial cell types. It switches the potential of neural stem cells from generating motor neurons to the differentiation into serotonergic neurons and in later stages into oligodendrocyte precursors (Dias et al., 2014). In the dorsal telencephalon, TGFβ has a similar temporal restricted potential to instruct neurogenesis. It induces neuronal differentiation of a subset of neural progenitor cells during the late phase of neurogenesis *in vitro* (Vogel et al., 2010; Wahane et al., 2014; Vezzali et al., 2016). However, in the early phase of neurogenesis, TGFβ-mediated neuronal differentiation is hampered by the presence of FOXG1 in neural progenitor cells (Seoane et al., 2004; Siegenthaler and Miller, 2005; Siegenthaler et al., 2008; Vezzali et al., 2016). Thus, TGFβ mediated control of differentiation underlies temporally and spatially restricted transcriptional programs.

Astrocyte development is controlled by a variety of signaling pathways, such as Notch- (Chambers et al., 2001; Tanigaki et al., 2001), ciliary neurotrophic factor- (CNTF) (Johe et al., 1996), janus kinase and signal transducer and activator of transcription- (JAK-STAT) (Bonni et al., 1997; Rajan and McKay, 1998) as well as bone morphogenic protein (BMP)-signaling (Gross et al., 1996; Mehler et al., 2000). TGFβ-signaling is also involved in astrocyte development, where it induces differentiation of RGCs into astrocytes *in vitro* and *in vivo* (Stipursky and Gomes, 2007; Stipursky et al., 2012, 2014). In primary astrocyte cultures, TGFβ reduces proliferation induced by basic fibroblast growth factor (bFGF), epidermal growth factor (EGF), plateled-derived growth factor (PDGF), interleukin-1α (IL-1α) and IL-2. However, in the absence of these mitogens TGFβ has no effects on proliferation (Flanders et al., 1993; Hunter et al., 1993). Moreover, TGFβ1 induces morphological changes, colony formation and increases GFAP-expression in primary cultures of entire mouse hemispheres (Flanders et al., 1993; De Sampaio e Spohr et al., 2002). Understanding how TGFβ affects astrocyte development and function is of clinical relevance as overproduction of TGFβ1 from astrocytes is associated with cerebrovascular degeneration resulting in an Alzheimer’s disease-like phenotype (Wyss-Coray et al., 2003).

The identification of regionally specific astrocyte functions has fostered new concepts of specialized and heterogeneous subtypes of astrocytes (Schitine et al., 2015; Tabata, 2015). Thus, paralleling neurogenesis, astrogenesis also underlies temporal and/or spatial heterogeneity. Cortical astrocytes were formerly distinguished as being fibrous or protoplastic according to morphology and GFAP-expression levels (Raff et al., 1983; Miller and Raff, 1984). Today, astrocyte diversity is described by distinct clonal origins and regional localization (Magavi et al., 2012; Tsai et al., 2012; Garcia-Marques and Lopez-Mascaraque, 2013), different expression patterns of astrocytic proteins (Raff et al., 1983; Miller and Raff, 1984; Emsley and Macklis, 2006; Hochstim et al., 2008; Zeisel et al., 2015), specific support or regulation of surrounding cells (Iino, 2001; Song et al., 2002; Panatier et al., 2006; Gourine et al., 2010; Saab et al., 2012; Molofsky et al., 2014), and specialized response to external signals (Tsai et al., 2012; Martín-López et al., 2013). A recent study proposed two different astrocyte populations in the cerebral cortex, distinguished by expression of GFAP and MFGE8 (Zeisel et al., 2015). The secreted protein MFGE8 is mainly expressed by astrocytes in the central nervous system (CNS) (Boddaert et al., 2007; Cahoy et al., 2008; Fuller and Van Eldik, 2008; Kranich et al., 2010; Fricker et al., 2012). During CNS injury and disease, MFGE8 is involved in microglia-mediated removal of stressed or injured neurons (Fuller and Van Eldik, 2008; Fricker et al., 2012; Neher et al., 2013; Neniskyte and Brown, 2013; Liu et al., 2015).

In this study, we applied quantitative proteomics after stable isotope labeling with amino acids in cultures (SILAC) of neural cells from the telencephalon of mice carrying a FOXG1-cre driven deletion of TGFBR2 (Tgfbr2-cKO). We identified that mainly proteins specific for astrocytes were altered in the Tgfbr2-cKO. We focused on GFAP and MFGE8, which were oppositely regulated and explored heterogeneous subpopulations of astrocytes in the dorsal (DT) and ventral telencephalon (VT) with regard to these proteins. We revealed that distinct astrocyte populations expressed MFGE8 or GFAP in the DT and VT and that they responded differently to TGFβ stimulation. Finally, we provide evidence that MFGE8- and GFAP-expressing astrocytes partly originated from FOXG1-expressing progenitor cells.

## Materials and Methods

Detailed description of methods is provided in the Supplementary Methods.

### Mouse Strains and Genotyping

The animal welfare committees of the University of Freiburg and local authorities approved all animal experiments, registered under the license X11/09S, X14/04H and G14/096. The following mouse lines have been used: FOXG1-cre (Hébert and McConnell, 2000), Tgfbr2-floxed (Chytil et al., 2002), Rosa26-Stop-EYFP (Srinivas et al., 2001), ALDH1L1-EGFP [Tg(Aldh1l1 EGFP)OFC789Gsat/Mmucd (Gong et al., 2003)]. For isolation of cells from WT mice, time pregnant NMRI mice and embryos at E13.5 were used. Genotyping PCRs are described in Supplementary Methods.

### SILAC, Sample Preparation, Mass Spectrometry and Data Analysis

Embryonic E13.5 WT NMRI or mutant cells deriving from the entire telencephalon were cultured as described above, with the exception that custom made neurobasal medium lacking lysine and arginine (Life Technologies, Darmstadt, Germany) but additionally supplemented with Lys4/Arg6 or Lys8/Arg10 (0.398 mM arginine and 0.798 mM lysine, CLM-2265-H-0.5, CNLM-539-H-0.5, CNLM-291-H-0.5, DLM-2640-0.5, ULM-8347-0.1, ULM-8766-01, Euriso-Top, Saarbrücken, Germany) was used. Tgfbr2-cKO and control cells were cultured until DIV12, ensuring a virtually complete labeling (Supplementary Figures S1B,C). The complete protocol with details on cell lysis, mass spectrometry, quantification and analysis is given in Supplementary Methods.

### Immunoblotting and Densitometric Analysis

Cells were harvested in RIPA buffer with complete Protease inhibitor cocktail (Roche) and snap frozen at -80°C. Cell lysates were thawed, incubated for 30 min on ice, triturating every 10 min 20 times and cell debris were removed by centrifugation. Protein concentrations were determined photometrically with Bio-Rad Protein Assay Dye Reagent Concentrate (#500-0006, Bio-Rad). Concentration was adjusted and samples were prepared for SDS-PAGE by addition of Laemmli-buffer and 5 min boiling at 95°C. SDS-PAGE was performed with 8 or 10% polyacrylamide-gels and proteins were transferred to PVDF membranes (Trans-blot Turbo Transfer Pack, Bio-Rad) using the Trans-Blot Turbo (Bio-Rad). Membranes were washed 5 min in TBST (TBS with 0.1% Tween 20) and incubated for 1 h in 5% BSA/TBST. Incubation with primary antibodies was performed over night at 4°C in 5% BSA/TBST. Before and after incubation with the second HRP (horseradish-peroxidase)-coupled antibody, membranes were washed three times with TBST. Membranes were detected with the Femto reagent (Thermo Scientific) using the LAS ImageQuant System (GE Healthcare, Little Chalfont, United Kingdom). GAPDH was used as a loading control in all experiments. The following primary and secondary antibodies were used: MFGE8 (goat, 1:1000, #AF2805, R&D Systems), GFAP (mouse, 1:1000, MAB360, Chemicon International), MAP2 (rabbit, 1:1000, ab32454, Abcam, Cambridge, United Kingdom), ALDH1L1 (rabbit, 1:500, Abcam), GAPDH (mouse, 1:5000, ab8245, Abcam), Nestin (mouse, 1:1000, ab6142, Abcam), STAT3 (rabbit, 1:1000, #9132, CST), P-STAT3 (rabbit, 1:2000, #9145, CST), anti-goat-HRP (donkey, 1:5000, sc-2020, SCBT), anti-rabbit-HRP (goat, 1:10000, 115-035-003, Dianova, Hamburg, Germany), donkey-anti-mouse-HRP (goat, 1:10000, 111-005-003, Dianova).

Densitometric analyses were done with FIJI (ImageJ). Values were normalized to GAPDH. Afterwards, treated or Tgfbr2-cKO conditions were normalized to respective control conditions. Graphics and statistical analyses were done with GraphPad Prism. Originals of the represented immunoblots for Figures 4, 5 are shown in Supplementary Figures S7, S8.

### Statistical Analyses

Mass spectrometry analysis was performed with the Perseus software employing a one-sample *t*-test.

The GraphPad Prism software was used for statistical analyses. Immunoblot results were normalized to GAPDH and to the respective control and a one-sample *t*-test was applied. ELISA data and cell countings were compared by an unpaired Student’s *t*-test. Values in bar charts were illustrated as an average with the standard error of the mean (SEM).

The respective statistical analyses and biological replicates are included in the figure legends.

## Results

### Quantitative Proteomics Reveals Altered Astrocyte Protein Levels in Telencephalic Cells of Tgfbr2-cKO

**FIGURE 1:**
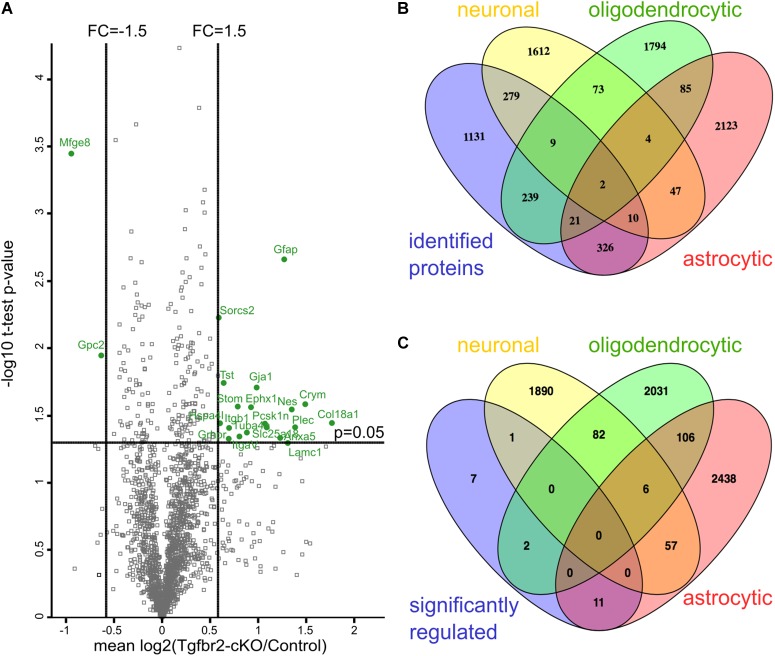
Quantitative proteomics reveals differential expression of astrocyte proteins after neural deletion of the Tgfbr2. **(A)** Scatterplot of identified proteins. Proteins with more than 1.5 fold change alteration and with a *p*-value < 0.05 in one-sample *t*-test are shown in green. Results are shown as mean values of the log2(fold change) of at least two out of four experiments. FC: fold change; one-sample *t*-test with cut-off at *p* = 0.05. **(B)** Comparison of all identified proteins of the proteome with gene arrays from Cahoy et al. (2008) demonstrate that proteins from the three major cell lineages of the CNS were identified. **(C)** Comparison of 21 significant differentially altered proteins with the gene arrays from Cahoy et al. (2008) shows 11 astrocyte-specific proteins.

To study the impact of TGFβ-signaling during neural development of the forebrain, we used the conditional mouse mutant (Foxg1^cre/+^;Tgfbr2^flox/flox^, in short Tgfbr2-cKO) recently characterized by Hellbach et al. (2014). To reveal global differences between the proteome of Tgfbr2-cKO and wildtype (WT) forebrains we applied SILAC and quantitative proteomics (Ong et al., 2002; Ong and Mann, 2006) with cultured neural progenitor cells isolated from E13.5 entire telencephalon. We used two different combinations of heavy lysines and arginines [Lys4/Arg6 and Lys8/Arg10 (Supplementary Figure S1A)] as described previously (Zhang et al., 2011). To monitor complete incorporation of labeled amino acids, we cultured forebrain cells until day *in vitro* (DIV) 6 or DIV12 and determined labeling efficiencies at these time points. We achieved virtually complete incorporation of heavy amino acids into WT telencephalic cells at DIV12 (Supplementary Figures S1B,C). Henceforth, we used this experimental set up to compare the global proteomes of primary neural Tgfbr2-cKO and WT cells from E13.5 telencephalon. By mass spectrometry we identified 2023 proteins, which contained at least two unique peptides and were present in at least two out of four independent biological replicates (Figure 1A).

**FIGURE 2:**
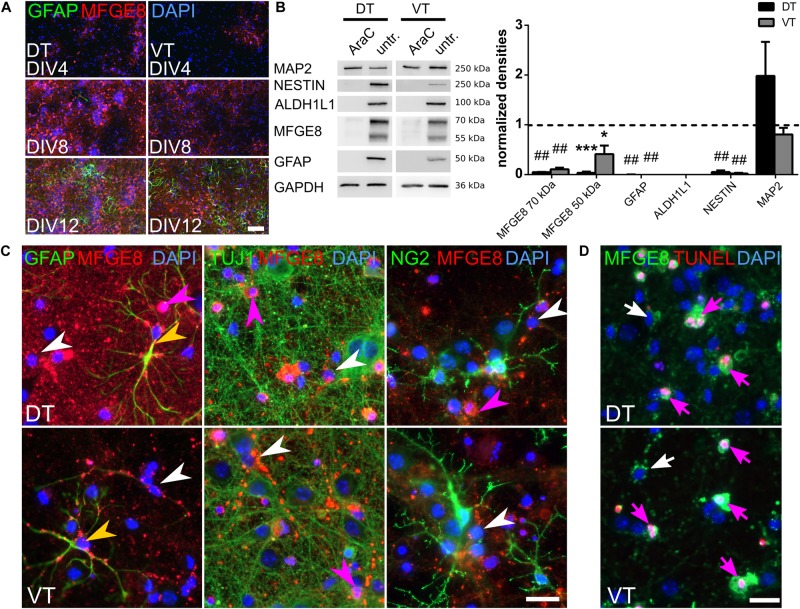
MFGE8 derives only from proliferating forebrain cells and localizes around GFAP fibers, but is not detectable in neurons or oligodendrocytes. **(A)** MFGE8- and GFAP-expression increases with differentiation of the cells in culture. Representative pictures of MFGE8 and GFAP stained DT and VT cells at DIV4, DIV8, and DIV12, scale bar: 200 μm, *n* = 3. **(B)** Left panel: representative immunoblots from AraC treated and untreated E13.5 DT and VT cells harvested at DIV12. MFGE8 is reduced by AraC treatment along with the astrocyte markers ALDH1L1, GFAP and the progenitor marker NESTIN. MAP2 levels are similar between AraC and untreated conditions. Right panel: densitometric analyses of the immunoblots. Mean with SEM; ^∗^*p* < 0.05; ^∗∗∗^*p* < 0.001; ##*p* < 0.0001; one-sample *t*-test; *n* = 3 (ALDH1L1), *n* = 4 (MFGE8, MAP2, NESTIN), *n* = 6 (GFAP). **(C)** Immunocytochemistry (ICC) from WT E13.5 DIV12 DT and VT cells confirms localization of MFGE8 puncta around astrocytic GFAP fibers (yellow arrowheads), but not around neuronal (TUJ1) or oligodendrocyte (NG2) processes. MFGE8 puncta appear also in cells with astrocyte morphology without detectable GFAP-positive staining (white arrowheads). Magenta arrowheads indicate MFGE8 opsonized dying cells. Scale bar: 20 μm, *n* = 3. **(D)** MFGE8 opsonized cells undergo cell death as shown by TUNEL staining (magenta arrows). White arrows indicate viable cells with MFGE8 puncta. Scale bar: 20 μm, *n* = 3.

To determine the cellular origin of the detected proteins, we compared all identified proteins with transcriptomes from either neurons, astrocytes or oligodendrocytes (Cahoy et al., 2008). According to this comparison, we identified similar numbers of proteins from these three neural lineages in our proteomic data set (Figure 1B). Thus, cells from all three lineages were present after DIV12 in the culture system and had incorporated heavy amino acids. Immunofluorescence analysis confirmed presence of neurons, astrocytes and NG2-(chondroitin sulfate proteoglycan 4) expressing oligodendrocyte precursors in these cultures (Figure 2C and Supplementary Figures S2D,E). Applying a one-sample *t*-test and a cut off of ±1.5 fold change, we shortlisted 21 candidate proteins, which had significantly altered expression levels in the Tgfbr2-cKO-derived proteome (Figure 1A and Table 1). Out of these 21 differentially expressed proteins, 11 proteins originated from astrocytes (Figure 1C). The two most significantly altered proteins were GFAP, which was 2.41 fold increased, and MFGE8, which was 1.91 fold less abundant in cells from Tgfbr2-cKO compared to WT. As expression of both genes classifies different astrocyte subtypes (Zeisel et al., 2015), we subsequently focused our analyses on these two proteins with regard to TGFβ-signaling in the developing telencephalon and astrocyte diversity.

**Table 1:** List of differentially regulated proteins of the Tgfbr2-cKO proteome.

Protein names	Gene names	Log2(FC)	SEM	*t*-test	Sequence coverage [%]	Mol. weight [kDa]
				*p*-value		
Lactadherin	Mfge8	-0.94	0.05	0.00035	37.1	47.17
Glial fibrillary acidic protein	Gfap	1.27	0.13	0.00215	63.8	49.36
VPS10 domain-containing receptor SorCS2	Sorcs2	0.59	0.05	0.00583	3.1	128.90
Glypican-2;Secreted glypican-2	Gpc2	-0.63	0.11	0.01107	20.4	62.36
Thiosulfate sulfurtransferase	Tst	0.64	0.13	0.01777	21.9	33.47
Gap junction alpha-1 protein	Gja1	0.98	0.14	0.01924	15.2	43.00
Ketimine reductase mu-crystallin	Crym	1.50	0.36	0.02566	47.3	33.52
Erythrocyte band 7 integral membrane protein	Stom	0.79	0.19	0.02676	37	31.38
Epoxide hydrolase 1	Ephx1	0.93	0.23	0.02699	42.6	52.58
Nestin	Nes	1.36	0.34	0.02817	19.3	207.12
Collagen alpha-1(XVIII) chain;Endostatin	Col18a1	1.77	0.34	0.03552	4.5	182.29
Heat shock 70 kDa protein 4L	Hspa4l	0.60	0.16	0.03559	21.1	94.38
ProSAAS	Pcsk1n	1.07	0.06	0.03597	14	27.27
Plectin	Plec	1.38	0.39	0.03772	23.1	513.73
Mitochondrial glutamate carrier 2	Slc25a18	1.09	0.22	0.03779	16.3	33.30
Integrin beta-1	Itgb1	0.70	0.20	0.03830	14.7	88.23
Tubulin alpha-4A chain	Tuba4a	0.88	0.26	0.04158	43.3	49.92
Integrin alpha-V	Itgav	0.81	0.18	0.04434	7.7	111.51
Annexin A5	Anxa5	1.23	0.37	0.04563	54.5	35.75
Glyoxylate reductase/hydroxypyruvate reductase	Grhpr	0.70	0.16	0.04643	11.6	35.33
Laminin subunit gamma-1	Lamc1	1.31	0.30	0.04929	5.1	177.19


### MFGE8 Is Expressed by Distinct Types of Telencephalic Astrocytes

MFGE8 was significantly reduced in the proteome of cultured Tgfbr2-cKO neural cells (Figure 1A). We did not detect strong expression of MFGE8 in the neuroepithelium of E13.5 Tgfbr2-cKO and WT forebrains *in vivo*. At this developmental time point MFGE8 mainly localized near isolectin B4 (IB4)-positive blood vessels (Supplementary Figure S2A). To specify the exact location of MFGE8 around the blood vessels *in vivo*, we analyzed co-localization of MFGE8 with IB4 (endothelial cells), platelet-derived growth factor receptor beta (PDGFRb) (pericytes) and pan-Laminin (basal lamina). The immunostainings together with electron microscopy analyses indicated that MFGE8 localized between endothelial cells and pericytes, where it co-localized with the basal lamina (Supplementary Figures S2B,C). Several reports describe astrocytes as the major source of MFGE8 in the CNS (Boddaert et al., 2007; Cahoy et al., 2008; Fuller and Van Eldik, 2008; Kranich et al., 2010; Fricker et al., 2012). We thus speculated that our *in vitro* cell culture contained astrocytes, which accounted for the high expression levels of MFGE8. We therefore characterized the cellular composition of the *in vitro* cultures from DT and VT after DIV12 (Supplementary Figures S2D,E and Figure 4I). The cultures contained a heterogenous mixture of different cell types with approximately 30% HuC/D-positive neurons, 5–6% NG2-positive oligodendrocytes, 3% (DT) or 1% (VT) TBR2-positive neural progenitor cells, 8–10% GFAP-positive astrocytes, 39% (DT) or 34% (VT) MFGE8-positive cells and 11% (DT) or 17% (VT) TUNEL-positive apoptotic cells. IB4-positive endothelial cells and microglia were rarely detectable and therefore not quantified. We concluded that the E13.5-derived neural progenitors differentiated into neurons, oligodendrocytes and astrocytes during the 12DIV and that these long-term cultures therefore expressed detectable levels of glial proteins, such as MFGE8.

To monitor the differentiation from progenitors into the astrocytic cell lineage, we followed the expression of MFGE8 and GFAP in cultures from E13.5 DT and VT at DIV4, 8, and 12 using immunocytochemistry (ICC) stainings. Both MFGE8- and GFAP-expressing cells increased until DIV12 (Figure 2A), indicating that the detected MFGE8 was of astrocytic origin. To further confirm the astrocytic origin of MFGE8 we interfered with cell proliferation using arabinofuranosyl cytidine (AraC), which diminished neural progenitors and astrocytes in the E13.5-derived WT cell cultures. Using immunoblotting and densitometric analyses, we observed a significant reduction of the astrocyte markers GFAP and aldehyde dehydrogenase 1 family, member L1 (ALDH1L1), as well as reduced MFGE8 levels after suppressing cell proliferation compared to untreated controls. Expression of the progenitor marker NESTIN was also reduced, but the neuronal protein microtubule-associated protein 2 (MAP2) was expressed at equal levels in both conditions (Figure 2B). To further confirm an astrocytic origin of MFGE8, we analyzed co-localization of MFGE8 with markers for neurons, astrocytes and oligodendrocytes with immunocytochemistry (ICC) in our culture system. As we observed differences in the secretome of Tgfbr2-cKO between the DT and VT (Hellbach et al., 2014), we assessed cells derived from both regions separately. MFGE8 localized in puncta in somata and along processes of GFAP-positive cells (Figure 2C, yellow arrowheads). These patterns did neither appear around tubulin beta 3 class III (TUJ1/TUBB3)-expressing neurons, nor around NG2-expressing oligodendrocytes (Figure 2C). Endothelial cells or microglia [detected by either IB4 or ionized calcium binding protein 1 (IBA1) and IB4 co-expression] were not present in significant numbers in these cultures and could hence be excluded as a source of MFGE8 (data not shown). Altogether, the findings from immunoblotting and -stainings suggested that astrocytes were the primary source of MFGE8 in the DT and VT cultures.

However, we observed strong MFGE8 staining, which co-localized also with cells that often had a fragmented nucleus (Figure 2C, magenta arrowhead). We hypothesized that these signals derived from dying cells, which were opsonized by MFGE8, as this is one of its known functions (Hanayama et al., 2002). We confirmed this observation by co-localization of TUNEL staining signals with MFGE8 opsonized cells (Figure 2D, magenta arrow).

**FIGURE 3:**
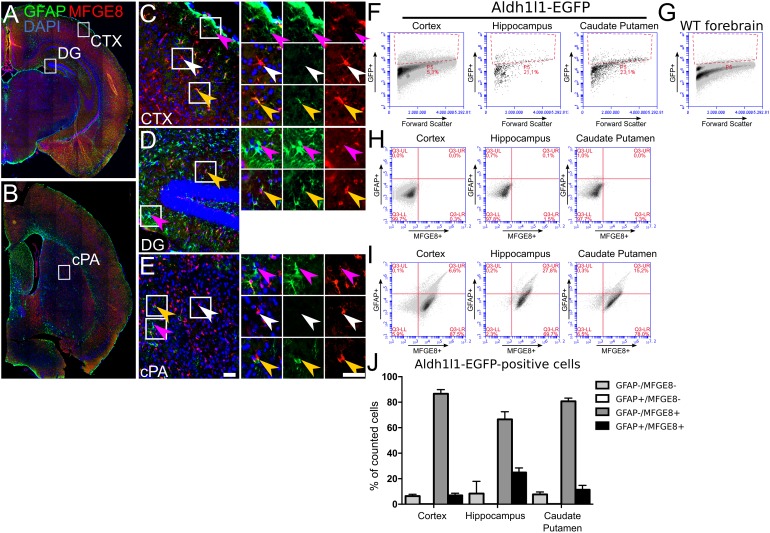
Astrocytes show distinct patterns of MFGE8- and GFAP-expression in adult mouse telencephalon. MFGE8 and GFAP astrocytes localization in coronal sections of 6 week old mouse forebrain, **(A)** caudal and **(B)** rostral sections. Magnifications of **(C)** cerebral cortex (CTX), **(D)** dentate gyrus (DG) and **(E)** caudate putamen (cPA) demonstrate that GFAP astrocytes (magenta arrowheads) reside mainly in glia limitans, dentate gyrus and caudate putamen. The cerebral cortex but also the caudate putamen contain large numbers of MFGE8 astrocytes (white arrowheads). MFGE8+/GFAP+ astrocytes (yellow arrowheads) appear mainly in hippocampus and near blood vessels in the cortical plate and in the caudate putamen. Scale bar: 50 μm, *n* = 3. **(F)** Flow cytometry gating for GFP-positive and **(G)** -negative cells was established using cells obtained from ALDH1L1-EGFP transgenic mice for the cerebral cortex, hippocampus and caudate putamen, and WT adult forebrain. **(H)** Analysis of MFGE8 and GFAP cell populations from three different brain regions with flow cytometry using only the secondary antibodies to set the background level given by unspecific binding in cortical tissue, hippocampus and caudate putamen. **(I)** Flow cytometry analysis from the same brain regions as in **(F)** using primary antibodies against GFAP and MFGE8. Three populations (GFAP+, MFGE8+ and GFAP+/MFG8+) were observed after gating and present in all brain regions. **(J)** Quantification of independent flow cytometry experiments showing presence of three subtype populations in all brain regions. Less than 1% ALDH1L1+/GFAP+/MFGE8- astrocytes were detected in the analyzed brain regions. Mean with SEM, *n* = 3.

MFGE8 puncta were also observed in cells with astrocyte morphology that were negative for GFAP (Figure 2C, white arrowhead). This observation supported recent findings from single-cell RNA sequencing showing that MFGE8- and GFAP-expression marked different subtypes of astrocytes in the cerebral cortex (Zeisel et al., 2015). Extending the data of Zeisel et al. (2015), our data showed that *in vitro* not only neural progenitors from the DT, but also from the VT, differentiated into these two subtypes of astrocytes (Figure 2C). *In vivo*, immunostainings of adult brain sections revealed the presence of GFAP-positive astrocytes in the glia limitans of the cerebral cortex and in proximity of the ventricles. In contrast, MFGE8-expressing astrocytes resided mainly in the cortical plate (Figures 3A,C, magenta and white arrowheads, respectively). We identified also small numbers of MFGE8/GFAP double-positive astrocytes within the cortical plate (Figures 3A,C, yellow arrowheads). In contrast, astrocytes in the hippocampus either co-expressed MFGE8 and GFAP, or GFAP alone, whereas single-positive MFGE8 astrocytes were hardly detectable (Figures 3A,D). The caudate putamen, the derivative of the VT, contained primarily MFGE8 astrocytes. GFAP and GFAP/MFGE8 double-positive astrocytes localized primarily near the ventricles and vessels (Figures 3B,E). The different astrocyte fractions of MFGE8 and GFAP single- and double-positive cells were already detectable at the neonatal P0 and juvenile P21 stage (Supplementary Figure S3A). We hypothesized that astrocyte heterogeneity with regard to MFGE8- and GFAP-expression might be established during development.

As we observed partial overlap of MFGE8- and GFAP-expression, which also seemed to vary in different anatomical locations, we determined co-expression of MFGE8 and ALDH1L1 or S100B. The respective immunostainings showed a large overlap of MFGE8 with these two astrocytes markers *in vivo*, but also highlighted astrocytes that did not co-express MFGE8 (Supplementary Figure S3B). To determine the fraction of MFGE8, GFAP and MFGE8/GFAP astrocytes among ALDH1L1 astrocytes in different brain regions in a quantitative approach, we sorted the ALDH1L1 astrocyte population from ALDH1L1-EGFP mice by applying flow cytometry (Figures 3F,G). We determined MFGE8- and GFAP-positive cells from the ALDH1L1-EGFP expressing cells and observed that the majority of the astrocytes was MFGE8 single-positive. Only a small fraction was MFGE8/GFAP double-positive in all analyzed brain regions. The fraction of GFAP single-positive cells was very small (<1%) (Figures 3H–J). Moreover, MFGE8 did not co-localize with NG2, OLIG2 (oligodendrocyte transcription factor 2), or IBA1 in adult hippocampus or cortex (Supplementary Figure S3C), supporting the *in vitro* data (Figure 2C), which excluded oligodendrocytes and microglia as a source of MFGE8-expression.

We concluded that astrocytes are the primary source of MFGE8 *in vitro* in the DT and VT cultures, as well as *in vivo* in the adult brain. Furthermore, we confirmed that MFGE8- and GFAP-expression defined distinct subtypes of astrocytes not only in the cerebral cortex, but also in the hippocampus and in the VT.

### TGFβ Suppresses MFGE8-Expression in Astrocytes From DT and VT

MFGE8 and GFAP marked different astrocyte populations in the DT and VT. As both proteins were altered in the Tgfbr2-cKO proteome (Table 1), we hypothesized that these astrocyte populations responded differently to the TGFBR2-deficiency. We aimed to elucidate in more detail how TGFβ affected MFGE8- and GFAP-expressing astrocytes. As localization of MFGE8 and GFAP *in vivo* suggested that astrocytes in the DT and VT were distinct subtypes, we cultured and examined primary cells from both regions individually.

**FIGURE 4:**
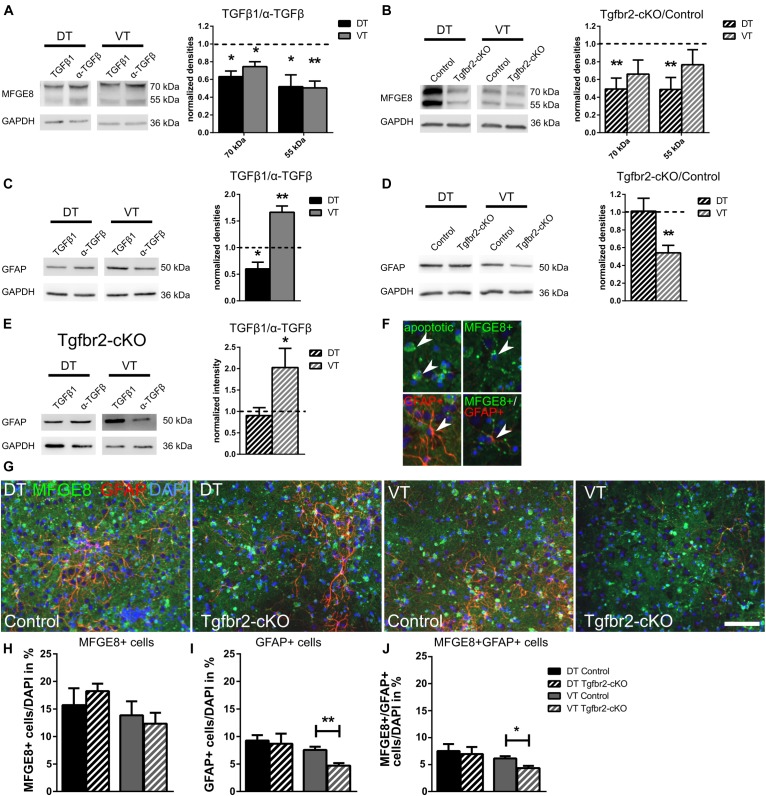
TGFβ-signaling reduces MFGE8-expression in DT and VT, and increases cell numbers of GFAP astrocytes specifically in VT. **(A)** Representative immunoblots (left panel) from WT DIV12 DT and VT cells treated with TGFβ1 (TGFβ1) or anti-TGFβ1,2,3 (α-TGFβ), and densitometric quantification (right panel). TGFβ treatment reduces MFGE8 levels in DT and in VT cells. *n* = 4. **(B)** Representative immunoblots (left panel) of DIV12 DT and VT cultures from Tgfbr2-cKO and respective controls, and densitometric quantification (right panel). MFGE8 levels are reduced in DT but not in VT cells. *n* = 9. **(C)** Representative immunoblots (left panel) from WT DIV12 DT and VT cells treated with TGFβ1 or anti-TGFβ1,2,3 and densitometric quantification (right panel). TGFβ treatment reduces GFAP levels in DT and increases GFAP levels in VT cells. *n* = 6. **(D)** Representative immunoblots (left panel) of DIV12 DT and VT cells from Tgfbr2-cKO and corresponding controls, and densitometric quantification (right panel). GFAP levels are decreased in cells from VT, but are unchanged in DT cells. *n* = 11 (DT), *n* = 7 (VT). **(E)** Representative immunoblots (left panel) of DIV12 DT and VT cells from Tgfbr2-cKO treated with TGFβ1 or anti-TGFβ1,2,3 and densitometric quantification (right panel). GFAP levels are unchanged in DT cells, but increased in VT cells of the Tgfbr2-cKO after TGFβ1 treatment. *n* = 10 **(F)** Examples of MFGE8 opsonized, apoptotic cells, MFGE8+ astrocyte, GFAP+ astrocyte and MFGE8+/GFAP+ astrocyte indicated by white arrowhead, used as counting reference for **(G)**. **(G)** Representative images of ICC for MFGE8 and GFAP used for quantification GFAP+, MFGE8+, and MFGE8+/GFAP+ astrocytes. Scale bar: 100 μm. **(H)** Quantification of the number of MFGE8+ astrocytes, **(I)** GFAP+ astrocytes and **(J)** MFGE8+GFAP+ astrocytes after immunofluorescence from DIV12 Tgfbr2-cKO and WT forebrain-derived astrocytes. **(A–E)** Mean with SEM; ^∗^*p* < 0.05; ^∗∗^*p* < 0.01: one-sample *t*-test. **(H–J)** Mean with SEM; ^∗∗^*p* < 0.01; unpaired student’s *t*-test; *n* = 4.

MFGE8 protein expression was monitored in WT neural cells treated for 10 days either with TGFβ1 to induce TGFβ-signaling, or with anti-TGFβ1/2/3 antibodies to inhibit endogenous TGFβ-signaling. Immunoblots revealed that TGFβ1 treatment of WT cells, both from DT and VT, resulted in decreased MFGE8 protein levels (Figure 4A). We concluded that TGFβ suppressed MFGE8 protein expression in astrocytes.

We next compared MFGE8-expression of Tgfbr2-cKO to WT cells from the DT and VT after 12DIV. Tgfbr2-cKO-derived DT astrocytes expressed reduced MFGE8 levels, but VT-derived Tgfbr2-cKO astrocytes did not express significantly different levels of MFGE8 (Figure 4B). Although these results seemingly indicated that astrocytes in the two different regions responded differently to TGFβ, our preceding experiment (Figure 4A) clearly showed that DT and VT cells were both repressing MFGE8-expression in response to TGFβ. This seemingly contradictory result is explainable by the observation that Tgfbr2-cKO DT cells excessively secreted TGFβ (Hellbach et al., 2014). Increased TGFβ levels in DT cultures decreased MFGE8 protein expression as expected from the preceding experiments (Figure 4A), in which treatment of DT cells with TGFβ1 reduced MFGE8 levels. A second, alternative explanation would be a different origin of the astrocytes, either from FOXG1-expressing or -negative progenitors. The persisting ability of the astrocytes to respond to the TGFβ-stimulus despite their origin from the Tgfbr2-cKO indicated that at least part of MFGE8-expressing astrocytes originated from neural progenitors that did not express FOXG1-cre to induce TGFBR2-deficiency in the DT.

In contrast, VT-derived cells of the Tgfbr2-cKO secreted similar levels of TGFβ compared to WT cells. Accordingly, we did not observe differences in MFGE8 protein expression between WT and Tgfbr2-cKO cells. Depletion of the TGFBR2 in VT cells might have increased MFGE8-expression compared to WT cells. As we did not observe increased MFGE8 levels, we hypothesized that VT-derived astrocytes originated from a FOXG1-cre independent cell lineage, similar to the MFGE8 astrocytes located in the DT.

Reduced cellular levels of MFGE8 protein might be caused by less protein production, excessive secretion of MFGE8 or altered numbers of astrocytes. We revealed a reduction of approximately 50% of MFGE8 levels in both, protein lysates (Figure 4B) and in conditioned medium, which contained secreted MFGE8 of Tgfbr2-cKO cells from DT (Supplementary Figure S4A). This finding indicated that reduced cellular levels of MFGE8 were caused by decreased protein expression and not by excessive secretion. Quantification of MFGE8-positive astrocyte numbers in DT and VT cultures from Tgfbr2-cKO and WT revealed no significant changes between both genotypes (Figures 4G,H). Thus, TGFβ suppressed MFGE8-expression, but not the generation of MFGE8-positive astrocytes.

Although MFGE8-opsonized, apoptotic cells were excluded from quantification of MFGE8-expressing astrocytes, we analyzed whether alterations in TGFβ-signaling changed the level of apoptosis, which would indirectly account for alterations in MFGE8-expression or numbers of MFGE8-expressing cells. Neither TGFβ-treatment nor the Tgfbr2-cKO altered the numbers of apoptotic, TUNEL-positive cells (Supplementary Figures S4B,C). Similarly, the fraction of apoptotic cells among MFGE8-positive cells remained constant upon TGFβ-treatment or in the Tgfbr2-cKO compared to controls (Supplementary Figures S4D,E). Thus, increased rates of apoptosis were not responsible for the altered levels of MFGE8 protein expression or numbers of MFGE8 astrocytes.

We concluded that TGFβ reduced MFGE8 protein expression in astrocytes derived from DT and VT, but that TGFβ did not alter numbers of MFGE8-expressing cells. Moreover, we hypothesized that MFGE8 astrocytes of the telencephalon derived at least in part from FOXG1-negative progenitors.

### TGFβ Reduces GFAP-Expression in Cells From DT, but Increases GFAP-Expression in Cells From VT

Previous reports showed that TGFβ induced astrocyte differentiation of RGC, which led to increased GFAP-expression and higher numbers of GFAP astrocytes *in vitro* and *in vivo* (Stipursky and Gomes, 2007; Stipursky et al., 2014). In contrast to these results was our observation that in the Tgfbr2-cKO proteome GFAP levels increased despite impaired TGFβ-signaling (Figure 1A and Table 1). We hypothesized that astrocyte heterogeneity and regional differences accounted for the observed different responses to TGFβ.

To elucidate putative regional differences with regard to GFAP-expressing cells, we cultured primary neural cells from E13.5 DT and VT separately and assessed GFAP protein levels at DIV12. Treatment of WT neural cell cultures with TGFβ1 or anti-TGFβ1,2,3 antibodies showed that TGFβ1 reduced GFAP levels significantly in DT cells, whereas it increased GFAP protein levels in VT cells (Figure 4C). Thus, TGFβ affected cells from DT oppositely compared to cells from VT with regard to GFAP protein expression.

Next, we assessed GFAP levels in Tgfbr2-cKO compared to WT cells. From the TGFβ treatments of WT cells we expected that GFAP protein levels were either increased in DT, and reduced or unchanged in VT cells of the Tgfbr2-cKO, respectively. However, DT cells from the Tgfbr2-cKO expressed similar levels of GFAP compared to WT cells. But, as expected, VT cells from the Tgfbr2-cKO expressed significantly less GFAP protein compared to WT controls (Figure 4D). We concluded that GFAP-expressing astrocytes comprised regional subtypes that responded differently to TGFβ.

GFAP protein levels in the Tgfbr2-cKO were unchanged in DT and decreased in VT compared to WT controls (Figure 4D). This finding seemed to be at odds with the increased GFAP levels that we identified in the Tgfbr2-cKO proteome (Figure 1A). In contrast to the immunoblots from Figure 4D, we determined the Tgfbr2-cKO proteome from a mixture of DT and VT cells. DT cells from the Tgfbr2-cKO excessively secreted TGFβ (Hellbach et al., 2014), and the Tgfbr2-cKO contained a cell population which did not derive from FOXG1-expressing progenitors and thus did not have the deletion of the TGFBR2, as shown above. Based on these findings, we hypothesized that GFAP-expressing astrocytes from the VT derived from a non-FOXG1-expressing progenitor, and that TGFβ derived from Tgfbr2-cKO DT cells induced GFAP protein expression in VT cells in the cell cultures used for the proteome. To experimentally validate this hypothesis, we treated DT as well as VT cells from Tgfbr2-cKO with TGFβ1 or anti-TGFβ1,2,3 antibodies and determined the GFAP protein levels. As expected, GFAP levels in DT astrocytes did not change, but increased in VT cells from Tgfbr2-cKO (Figure 4E). This experiment confirmed that Tgfbr2-cKO cells from the DT were not responsive to TGFβ-signaling, whereas VT-derived astrocytes retained increased GFAP levels in response to a TGFβ stimulus. Thus, GFAP astrocytes of the DT derived from FOXG1-expressing progenitors, whereas ventrally allocated GFAP astrocytes originated at least partly from FOXG1-negative progenitors.

We next analyzed the number of GFAP-positive astrocytes in DT and VT cultures from Tgfbr2-cKO to determine if loss of TGFβ-signaling affected expression of GFAP or numbers of GFAP-positive astrocytes. In DT cell cultures the number of astrocytes was not altered between Tgfbr2-cKO and WT conditions, whereas VT cultures from Tgfbr2-cKO contained approximately 50% less GFAP-positive cells (Figures 4G,I). These results are in line with the reduced GFAP-expression levels detected in VT astrocytes of the Tgfbr2-cKO (Figure 4D).

As we revealed presence of MFGE8/GFAP double-positive cells *in vivo* in the P0, P21 and adult forebrain, we quantified the numbers of MFGE8/GFAP double-positive cells *in vitro* after deletion of the TGFBR2. We observed that the majority of GFAP-positive astrocytes *in vitro* expressed also MFGE8, and accordingly cultures from mutant animals contained significantly fewer MFGE8/GFAP double-positive cells compared to controls (Figure 4J). We concluded that TGFBR2-deficiency led to a reduction of GFAP-positive cells in VT cultures rather than influencing directly GFAP-expression.

### TGFβ-Signaling Acts Cell-Autonomously on GFAP-, but Not on MFGE8-Expression

**FIGURE 5:**
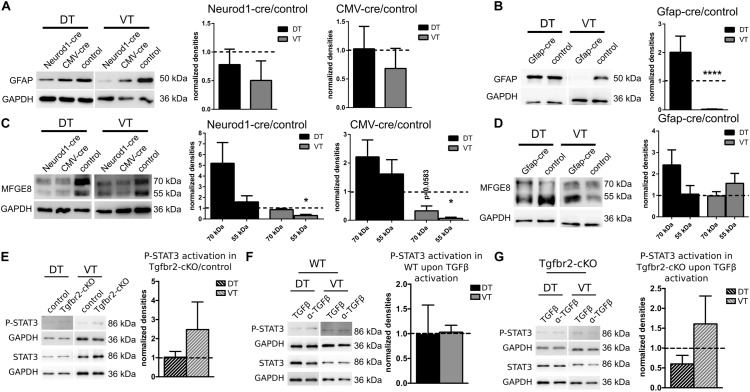
GFAP-expression changes cell-autonomously, MFGE8-expression non cell-autonomously, independent of JAK-STAT-signaling. **(A)** Representative immunoblots (left panel) and densitometric quantification (right panels) of DIV12 DT and VT cultures from Tgfbr2-cKO cells after expression of NEUROD1-cre and CMV-cre. GFAP levels remain unchanged. NEUROD1-cre: *n* = 8 (DT), *n* = 4 (VT); CMV-cre: *n* = 8 (DT), *n* = 5 (VT). **(B)** Representative immunoblots (left panel) and densitometric quantification (right panel) of DIV12 DT and VT cultures from Tgfbr2-cKO cells after expression of GFAP-cre. GFAP levels are decreased in VT. *n* = 7 (DT), *n* = 6 (VT). **(C)** Representative immunoblots (left panel) and densitometric quantification (right panels) of DIV12 DT and VT cultures from Tgfbr2-cKO cells after expression of NEUROD1-cre and CMV-cre. MFGE8 levels are reduced in VT only after deletion of TGFBR2 through NEUROD1-cre and CMV-cre. NEUROD1-cre: *n* = 8/6 (DT: 70 kDa/55 kDa), *n* = 3 (VT); CMV-cre: *n* = 8/7 (DT: 70 kDa/55 kDa), *n* = 3 (VT). **(D)** Representative immunoblots (left panel) and densitometric quantification (right panel) of DIV12 DT and VT cultures from Tgfbr2-cKO cells after expression of GFAP-cre. MFGE8 levels remained unaffected. *n* = 8/7 (DT: 70 kDa/55 kDa), *n* = 6 (VT). **(E)** Representative immunoblots and densitometric quantification showing levels of STAT3 phosphorylation in DIV12 DT and VT cultures from untreated Tgfbr2-cKO cells (*n* = 3). **(F)** TGFβ treated wild type cells [*n* = 3 (DT), *n* = 4 (VT)] **(G)** and TGFβ treated Tgfbr2-cKO cells, *n* = 3. GAPDH was used as loading control. Mean with SEM; ^∗^*p* < 0.05; ^∗∗^*p* < 0.01; ^∗∗∗∗^*p* < 0.0001: one-sample *t*-test.

We next investigated whether the altered GFAP- and MFGE8-expression upon TGFβ-signaling was mediated cell-autonomously or non-autonomously. Therefore, we deleted the TGFBR2 by virus-mediated delivery of CRE in cultured *Tgfbr2*-floxed neural cells isolated from E13.5 animals. CRE-expression was under control of GFAP-, NEUROD1-, or CMV-promoter, respectively. TGFβ-mediated increase in GFAP-expression was a cell-autonomous effect in VT cells, as TGFBR2-deficiency mediated by NEUROD1- or CMV-cre did not result in significant alterations of the GFAP-expression levels (Figure 5A). In contrast, astrocytic GFAP-cre mediated loss of TGFBR2-expression in VT-derived astrocytes led to nearly complete loss of GFAP protein expression. But DT-derived GFAP-positive astrocytes did not express significantly different levels of GFAP with or without intact TGFβ-signaling (Figure 5B).

On the other hand, GFAP-cre mediated TGFBR2-deficiency did not affect MFGE8-expression significantly (Figure 5C) compared to control cells. But neuronal, NEUROD1-cre and non-cell type specific CMV-cre mediated TGFBR2-deficiency decreased expression of the 55 kDa MFGE8-isoform significantly in the VT cells. But the DT cells were unaffected (Figure 5D). These experiments suggested that TGFBR2-expression in GFAP-positive cells did not affect MFGE8 levels, but that at least in part non cell-autonomous signals secreted from neurons in response to TGFβ-signals increased astrocytic MFGE8-expression.

As activation of the JAK-STAT pathway is integral during astrocyte differentiation we investigated P-STAT3 levels in cells of the Tgfbr2-cKO compared to controls, as well as after TGFβ-stimulation in WT or Tgfbr2-cKO cells (Figures 5E–G). Neither deletion of the TGFBR2 in the Tgfbr2-cKO nor stimulation with TGFβ led to significant changes in P-STAT3 levels.

In conclusion, impaired TGFβ-signaling decreased GFAP astrocytes in the VT in a cell-autonomous manner, whereas MFGE8-expression seemed to be controlled partly by secondary factors from other cells. The TGFβ-mediated effects seemed to be independent from activated JAK-STAT-signaling.

### FOXG1-Expressing Neural Progenitors of the Telencephalon Give Rise to Distinct Astrocyte Subtypes

The experiments above indicated that MFGE8- and GFAP-expressing astrocytes had distinct precursors, which probably differed with regard to FOXG1-expression. Hence, GFAP astrocytes from the DT derived from FOXG1-expressing progenitors, whereas VT-derived GFAP-expressing astrocytes originated from a different precursor subtype. We detected only sparse and faint co-expression of CRE with GFAP in cultures from FOXG1-cre animals (data not shown). Based on these findings we assumed that FOXG1 itself is not expressed by mature astrocytes, but only by their progenitors. To clarify whether DT and VT astrocytes originated from different progenitors that could be discriminated through FOXG1-expression in the pre-astrocytic developmental stage, we performed two different lineage-tracing experiments. First, we used an *in vitro* approach and transduced primary E13.5 cortical cells with a lentiviral reporter construct, which expressed mCherry in WT cells. Expression of CRE recombinase excised mCherry and activated GFP (Supplementary Figure S5A). After transduction of WT and FOXG1-cre expressing cells that were isolated from E13.5 DT and VT, we induced astrocyte differentiation by increasing serum levels. Cells were analyzed at DIV12 using ICC with anti-GFAP, -mCherry and -GFP antibodies. We identified a small fraction of cells that was double-positive for GFAP and GFP in FOXG1-cre expressing cells in DT and VT cultures. This finding provided evidence that progenitors from a FOXG1-expressing cell lineage differentiated into GFAP-positive astrocytes (Supplementary Figure S5B).

**FIGURE 6:**
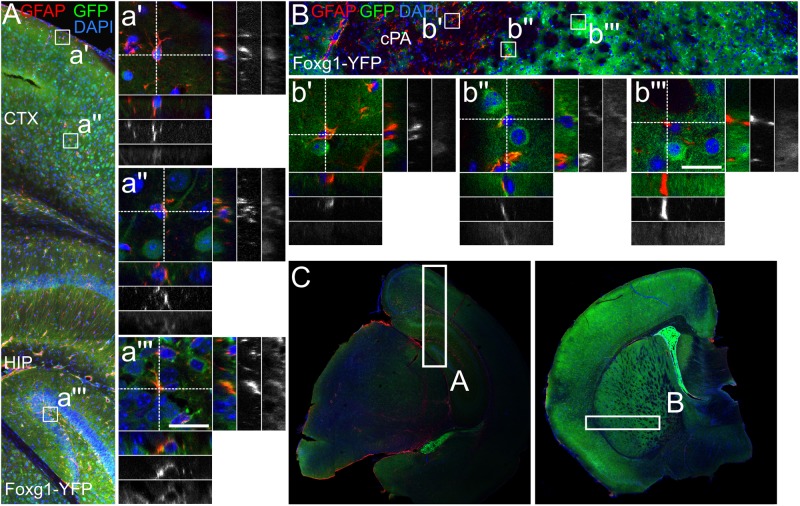
GFAP-positive astrocytes partially derive from FOXG1-cre expressing progenitors. Lineage tracing of FOXG1-expressing cells with a YFP reporter mouse line demonstrates that GFAP expressing astrocytes derive from FOXG1-expressing progenitors in **(A)** cerebral cortex (CTX) and hippocampus (HIP) and in **(B)** the caudate putamen (cPA). Magnifications show single GFAP- and YFP-positive (GFAP+/YFP+) cells in **(a’,a”)** cerebral cortex, **(a”’)** hippocampus and **(b”)** caudate putamen. In **(b’,b”’)** GFAP+/YFP-negative cells are illustrated in the caudate putamen. Scale bar: 10 μm, *n* = 3. **(C)** Overview images of representative rostral and caudal forebrain section after immunofluorescence for GFAP and GFP show the region analyzed in (A,B) as indicated.

To assess if astrocytes were a progeny of FOXG1-expressing neural precursors *in vivo*, we crossed Foxg1^cre/+^ mice with a reporter mouse (R26-stop-YFP). YFP-expression was activated in cells originating from a FOXG1-cre-expressing lineage. We used a cross-reacting anti-GFP antibody to visualize the YFP-signal. We first investigated GFAP astrocytes that expressed YFP in the cerebral cortex, hippocampus and in caudate putamen (Figures 6A–C). In the hippocampus and cerebral cortex we identified primarily GFAP+/GFP+ astrocytes (Figures 6a’–a”’), but no GFAP+/GFP- astrocytes. In the caudate putamen we determined GFAP+/GFP+ (Figure 6b”) and GFAP+/GFP- (Figures 6b’,b”’) astrocytes. These findings are in line with our observations *in vitro*.

**FIGURE 7:**
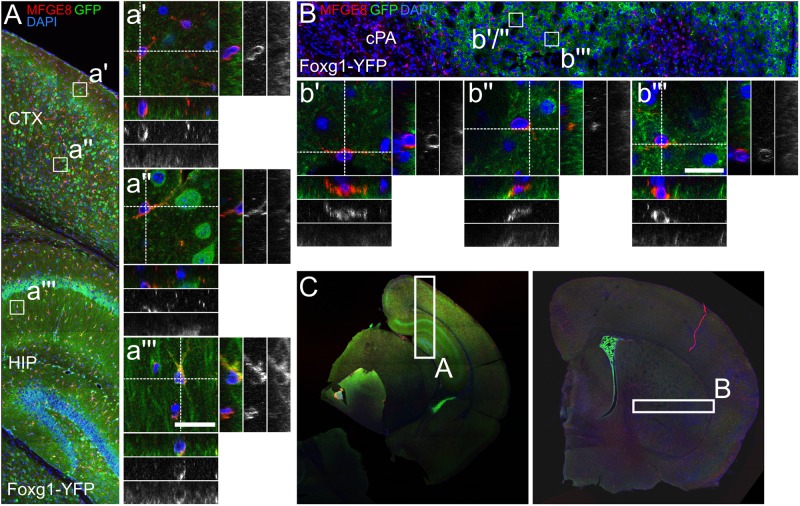
MFGE8-positive astrocytes partially originate from FOXG1-cre expressing progenitors. **(A)** Lineage tracing of FOXG1-expressing cells with a YFP reporter mouse line demonstrates that MFGE8 astrocytes can derive from FOXG1-expressing and other progenitors in **(A)** cerebral cortex (CTX) and hippocampus (HIP) and in **(B)** the caudate putamen (cPA). Magnifications show single MFGE8- and YFP-positive (MFGE8+/YFP+) cells in **(a”)** cerebral cortex, **(a”’)** hippocampus and **(b”,b”’)** caudate putamen. MFGE8+/YFP-negative cells are illustrated in **(a’)** the cerebral cortex and in **(b’)** the caudate putamen. Scale bar: 10 μm, *n* = 3. **(C)** Overview images of representative rostral and caudal forebrain section after immunofluorescence for MFGE8 and GFP show the region analyzed in (A,B) as indicated.

Next, we assessed if MFGE8 astrocytes were generated from FOXG1-expressing progenitors as well (Figures 7A–C). We identified MFGE8+/GFP+ astrocytes in cerebral cortex (Figure 7a”), hippocampus (Figure 7a”’) and caudate putamen (Figures 7b”,b”’). Moreover, we identified MFGE8+/GFP- astrocytes in cerebral cortex (Figure 7a’) and in caudate putamen (Figure 7b’).

**FIGURE 8:**
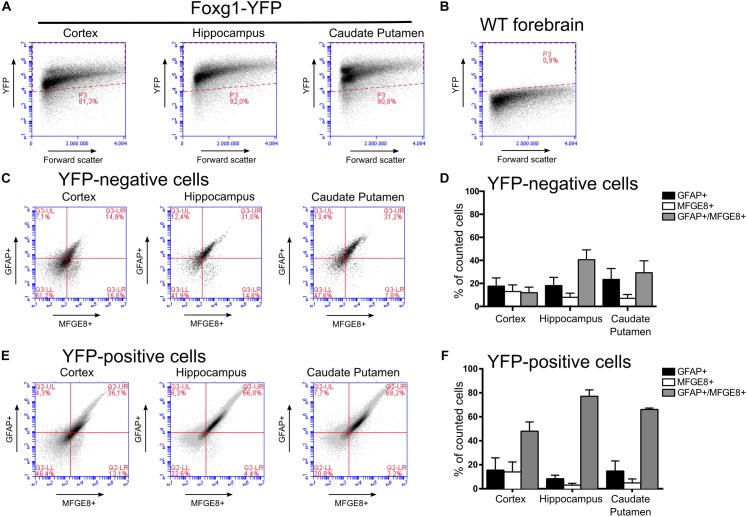
MFGE8 and GFAP-positive astrocyte derive from FOXG1-expressing and non-expressing progenitors in the cerebral cortex, hippocampus and caudate putamen. **(A)** Flow cytometry gating for YFP-positive and **(B)** -negative cells was established using cells obtained from Foxg1^cre/+^;R26-stop-YFP for the cerebral cortex, hippocampus and caudate putamen, and WT adult forebrain. **(C)** Analysis of MFGE8 and GFAP cell populations from three different brain regions with flow cytometry after gating YFP-negative cells. **(D)** Quantification of independent flow cytometry experiments as exemplified in **(C)**. Mean with SEM, *n* = 3. **(E)** Analysis of MFGE8 and GFAP cell populations from the three different brain regions with flow cytometry after gating YFP-positive cells. **(F)** Quantification of independent flow cytometry experiments as exemplified in **(E)**. Mean with SEM, *n* = 3.

Using flow cytometry we quantified the different fractions of GFAP- and MFGE8-expressing astrocytes originating from FOXG1-expressing (YFP-positive) or -negative (YFP-negative) progenitor cells (Figure 8A). We used WT forebrains to establish the gating for the YFP-positive and -negative cells of Foxg1^cre/+^; R26-stop-YFP forebrains (Figure 8B). We detected MFGE8+, GFAP+, and MFGE8+/GFAP+ astrocytes in cells derived from FOXG1-negative progenitor cells (Figures 8C,D) as well as in FOXG1-expressing (YFP-positive) progenitor cells (Figures 8E,F) in all three neuroanatomical regions that we analyzed. We concluded that FOXG1-expressing progenitors have the capacity to differentiate into astrocytes, but they are not the only source of MFGE8+, GFAP+ and MFGE8+/GFAP+ astrocytes in the telencephalon. All these lineage-tracing experiments suggested that FOXG1-expression at the pre-astrocytic development of the forebrain is an early hallmark of astrocyte heterogeneity.

## Discussion

Recent progress in neuroscience indicated that astroglia comprise a diversity of subtypes, which support and control various functions specific to their location and/or origin. It is for example of clinical interest to understand whether and how the heterogenous astrocyte population responds differently to brain injuries and inflammation (Götz et al., 2015; Ramos, 2016; Liddelow et al., 2017). It is therefore important to resolve the developmental and functional basis of astrocyte heterogeneity. Here, we expand the current knowledge of astrocyte diversity at three levels with regard to (1) expression of marker proteins MFGE8 and GFAP, (2) responsiveness to TGFβ, and (3) astrocyte subtypes as progeny of FOXG1-expressing neural stem cells.

We used SILAC and quantitative proteomics to determine cellular and molecular changes of neural cells from Tgfbr2-cKO. SILAC is routinely used with proliferating cell types, whereas studies of postmitotic cells suffer from incomplete labeling incorporation (Liao et al., 2008; Spellman et al., 2008; Zhang et al., 2011). Mathematically, five cell divisions lead to a labeling efficiency of more than 97% (Ong et al., 2002). But due to differentiation, the proliferation of primary neural progenitor cells is limited. Long cell culture periods or normalization to an internal labeling efficiency control circumvented the problem of incomplete labeling of postmitotic neurons (Liao et al., 2008; Spellman et al., 2008). Here, we applied a different strategy based on usage of two different combinations of heavy lysines and arginines (Lys4/Arg6 and Lys8/Arg10) (Zhang et al., 2011), which rendered our mass spectrometry analyses independent of remaining unlabeled peptides. We achieved virtually complete label incorporation after DIV12 and identified labeled proteins from neurons, astrocytes and oligodendrocytes. Our results provide evidence that the SILAC procedure is suitable for investigating the proteomes of primary neural cells including postmitotic neurons.

The majority of differentially altered proteins in the Tgfbr2-cKO proteome derived from astrocytes. Concomitant identification of neuronal and oligodendroglial proteins made it unlikely that labeling artifacts, introduced by higher proliferation rates of astrocytes or oligodendrocytes, accounted for this finding. Our mass spectrometry approach revealed that TGFBR2-deficiency reduced the expression level of astrocytic MFGE8. However, TGFβ-treatment also reduced levels of MFGE8. We previously revealed that the culture of TGFBR2-deficient cells contained increased levels of TGFβ (Hellbach et al., 2014), which accounted for reduced MFGE8 levels in cells that were not targeted by the expression of the Cre-recombinase. Our further analyses suggested that the levels of the 55 kDa isoform of astrocytic MFGE8 were decreased by TGFβ through a non cell-autonomous process, probably by signals deriving from neurons.

Based on our and other findings, we propose that TGFβ might be implicated in balancing MFGE8 levels, thereby protecting neurons from apoptotic elimination. TGFβ is secreted by various cell types after brain injury and activates astrocytes, microglia and neurons to induce anti-inflammatory responses (Zhu et al., 2002; Brionne et al., 2003; Dhandapani and Brann, 2003; Makwana et al., 2007; Doyle et al., 2010; Graciarena et al., 2013; Cekanaviciute et al., 2014a,b). As part of these responses, phagocytosis of apoptotic cells is performed primarily by activated microglia (Fuller and Van Eldik, 2008; Cheyuo et al., 2012; Fricker et al., 2012; Deroide et al., 2013; Neher et al., 2013; Neniskyte and Brown, 2013; Liu et al., 2015), which increase MFGE8 expression in response to TGFβ (Spittau, 2015). Elevated levels of MFGE8 lead to excessive removal of stressed neurons (Fricker et al., 2012; Neniskyte and Brown, 2013; Liu et al., 2015), and deletion of MFGE8 or blocking of its receptor attenuate neuronal loss by microglial phagocytosis *in vitro* and *in vivo* (Fricker et al., 2012; Neher et al., 2013; Neniskyte et al., 2014). Thus, the reduction of astrocytic MFGE8 in response to TGFβ might prevent neuronal stress and/or attenuate excessive phagocytosis of viable neurons.

We further found that GFAP astrocytes from the DT or VT reacted differently to TGFβ stimuli. GFAP-expression of VT astrocytes increased in a TGFβ-dependent, cell-autonomous manner. Others reported as well that TGFβ activates GFAP-expression and induces astrocyte differentiation of RGC *in vitro* as well as *in vivo*, with regional differences in the latter (Stipursky and Gomes, 2007; Stipursky et al., 2014). Presence of TGFβ induced morphological changes and colony formation (Flanders et al., 1993), activated GFAP-expression (De Sampaio e Spohr et al., 2002), but also attenuated proliferation of primary astrocytes in presence of other active signaling pathways (EGF, bFGF, PDGF, IL-1β, IL-2) (Flanders et al., 1993; Hunter et al., 1993). Together these findings suggest that the response of GFAP astrocytes to TGFβ stimuli are context-dependent. The time point, concentration of TGFβ as well as presence of other cells from or within different brain regions might be critical variables that account for the different observations regarding TGFβ-mediated GFAP-expression.

MFGE8- and GFAP-expressing astrocytes were seemingly different from each other, as they responded differently to TGFβ signaling. Interestingly, gene ontology analysis of the MFGE8 and GFAP astrocyte populations, based on their differential transcription of specific genes (Zeisel et al., 2015), suggested that MFGE8 astrocytes might affect for example endothelial cells, whereas GFAP astrocytes might influence neuronal differentiation (Supplementary Figure S6).

Astrocyte heterogeneity might originate from diverged development and/or depend on regional localization (Martín-López et al., 2013; Ramos, 2016). Diversification of neural stem cell progeny as observed within the neuronal lineage might also generate distinct functional astrocyte subtypes, suggesting that different progenitors give rise to different astrocytes (Pinto and Götz, 2007; Bayraktar et al., 2015). The results of the lineage tracing experiments using FOXG1-cre expression in Rosa-26-STOP-YFP support the view of different developmental origins of astrocytes, because we showed that MFGE8-, GFAP- and MFGE8-/GFAP-expressing astrocytes derived from FOXG1-expressing and non-expressing progenitor lineages. However, we did not reveal a distinct origin for a specific subtype. But FOXG1-expression in neural precursor cells led to the identification of different astrocyte subtypes specifically in the DT and VT. In addition, our findings suggest that not all precursor cells in the forebrain express FOXG1. Since FOXG1 expression was not observed in mature, adult astrocytes, we took advantage of the FOXG1-cre line. However, the recombination pattern of the line might differ between strains and loxP alleles, and recombination has been observed in cells within the CNS and other tissues without detectable levels of FOXG1 (Hébert and McConnell, 2000). CRE activity in non-FOXG1-expressing cells would result in false positive, FOXG1-derived astrocytes in our lineage tracing study. We cannot rule out completely that non-FOXG1-expressing cells recombined the reporter allele. However, our hypothesis that astrocytes originated from FOXG1-expressing and non-FOXG1-expressing cell lineages derived from the study of Tgfbr2-cKO animals. Thus, our conclusion of different astrocytic origins with regard to the FOXG1 lineage is based on two independent loxP alleles. We also transfected a plasmid carrying loxP alleles and observed astrocytes from the FOXG1-lineage *in vitro*. We therefore propose that the loxP alleles used in this study are stably reflecting FOXG1-cre activity.

Highly specialized types of astrocytes occur in all regions of the CNS. How these astrocytes obtain their specialization, whether this is specified intrinsically by their origin or rather extrinsically by surrounding cells or by secreted factors is not known in full detail yet.

This study provides evidence that different progenitors, with regard to allocation and FOXG1-expression, generate different astrocyte types, which (1) can be distinguished by MFGE8- and GFAP-expression and (2) by their response to TGFβ stimuli.

## Author Contributions

TV, KT, AV, and SW: design of the study and experiments, analyses and interpretation of data. TV, SW, and AV: wrote the manuscript. SW, FD, SH, SN, and AV: experimental setup and realization, analyses and interpretation of the data, and compilation of the figures. CS and JS: sharing of experimental resources, involved in experimental realization, and data analyses of mass spectrometry.

## Conflict of Interest Statement

The authors declare that the research was conducted in the absence of any commercial or financial relationships that could be construed as a potential conflict of interest.
