# Supplementary material for: TGFβ-Signaling and FOXG1-Expression Are a Hallmark of Astrocyte Lineage Diversity in the Murine Ventral and Dorsal Forebrain
Source: Front Cell Neurosci. 2018 Nov 28;12:448. doi: 10.3389/fncel.2018.00448 (PMC6282056; doi:10.3389/fncel.2018.00448)
Supplement: Supplementary file 1 [file Data_Sheet_1.docx]

Supplementary Methods:

**Primary neural cell culture of E13.5 telencephalon cells:**

Embryonic E13.5 dorsal and ventral telencephalon cells were dissected and collected in ice cold Hanks' Balanced Salt Solution (HBSS, Fisher Scientific, Schwerte, Germany) and dissociated in 0.25% Trypsin/EDTA (Fisher Scientific) at 37°C for 5 min. The dissociation was stopped by addition of NB-complete medium and 10% of the final volume foetal bovine serum (FBS, Fisher Scientific) and single cell suspension was obtained by trituration. Cells were collected by centrifugation at 200 rcf and resuspended in NB complete medium. Cells were cultured in NB-complete medium, consisting of Neurobasal medium (Fisher Scientific) supplemented with B27 (Fisher Scientific), L-glutamine (0.5 mM, Fisher Scientific), Penicillin-Streptomycin-Neomycin (PSN, Fisher Scientific), apo-transferrin (5 µg/ml, Sigma, München, Germany), superoxid-dismutase (0.8 µg/ml, Sigma) and glutathione (1 µg/ml, Sigma). Cells were always seeded on poly-ornithine (0.1 mg/ml, Sigma) and laminin (1 µg/ml, Sigma) coated dishes or glass cover slips. Cells for immunoblotting were cultured in 1.5 ml NB-complete in 6 well plates.

Cells from each Tgfbr2-cKO (and control) DT or VT were divided into 2 wells of a 6-well plate. Medium was changed at DIV6 and cells were cultured until DIV12. For experiments using WT NMRI 1.2 million DT cells or 1.5 million VT cells were seeded per well of a 6-well plate and cultured in 1.5 ml NB-complete. For treatments of Tgfbr2-cKO cells with TGFβ or anti-TGFβ1,2,3, 1/4 of the cells of each embryo deriving either from DT or VT was seeded in 3 wells of 12-well plates and were cultured in 1 ml NB-complete medium.

For treatment experiments, cells were treated on DIV2 with either 4 µM AraC, 5 ng/ml TGFB1 (PeproTech, Hamburg, Germany), or anti-TGFβ1,2,3 antibody (1 µg/ml, R&D Systems, Wiesbaden, Germany). At DIV7, medium that included the same concentration of additives was changed, and cells were harvested at DIV12 in RIPA (radioimmunoprecipitation assay) buffer (1% NP-40, 1% SDS, 0.5% sodium deoxycholate, 1 x PBS).

For immunocytochemistry, cells were seeded on glass coverslips and cultured in 0.5 ml NB-complete, with one medium change at DIV6 and fixation at DIV12 for 20 min with 4% PFA. 600,000 NMRI WT cells or 1/4 of the cells from each Tgfbr2-cKO (or control) VT or DT were seeded on 6 coverslips.

**Genotyping**

PCRs were performed from tail DNA, lysed in QuickExtract DNA Extra Solution (Biozym Scientific GmbH, Hessisch Oldendorf, Germany), using the following primers:

**Foxg1-cre:**

Bf1-F25: GCCGCCCCCCGACGCCTGGGTGATG

Bf1-R159: TGGTGTGGTGATGATGATGATGGTGATGCTG

Bf1-Rcre: ATAATCGCGAACATCTTCAGGTTCTGCGGG

Amplicon sizes: Foxg1-cre (~300 bp), Foxg1-WT (~200 bp)

**Tgfbr2-flox:**

LaLox: ACTTCTGCAAGAGGTCCCCT
8wa: TAAACAAGGTCCGGAGCCCA

Amplicon sizes: Tgfbr2-loxP (540 bp), Tgfbr2-WT (420 bp)

**Rosa26-stop-YFP:**

Rosa-YFP-1: AAAGTCGCTCTGAGTTGTTAT

Rosa-YFP-2: GCGAAGACTTTGTCCTCAACC

Rosa-YFP-3: GGAGCGGGAGAAATGGATATG

Amplicon sizes: Rosa26-stop-YFP cKO (320 bp), Rosa26-stop-YFP WT (600 bp)

**SILAC, sample preparation, mass spectrometry and data analysis:**

Cells were lysed in RIPA buffer supplemented with complete Protease inhibitor cocktail (Roche-Diagnostics, Mannheim, Germany), ultra sonicated 10 times 10 sec on/off with the BioRuptor Next Gen Sys (Diagenode, Seraing, Belgium) and cell debris were removed by centrifugation. Protein concentrations in cell lysates were quantified photometrically with Bio-Rad Protein Assay Dye Reagent Concentrate (#500-0006, BioRad, München, Germany). Equal amounts of protein from medium and heavy labeled cell lysates were mixed in a 1:1 ratio and 25 µg protein were separated by SDS-PAGE. The gel (Mini-PROTEAN® TGXT, 456-1084, BioRad) was stained with Coomassie solution and the lane was cut into 10 slices. Gel slices were destained with 30% acetonitrile in 100 mM ammoniumbicarbonate and dried with 100% acetonitrile. Proteins were digested in the gel by addition of 5 ng/µl trypsin in 100 mM ammoniumbicarbonate over night at 37°C. Peptides were extracted from the gel pieces using 5% formic acid followed by two times 100% ethanol. The peptides were desalted using C18 Stage Tips (Rappsilber et al., 2007).

In total four different biological replicates were prepared, including 1 label switch. LC/MS analysis was performed on an LTQ Orbitrap XL (Thermo Fisher Scientific, Bremen, Germany) either coupled to an Agilent 1200 (Agilent Technologies, Waldbronn, Germany) or an Eksigent 2D nanoflow-HPLC (AB Sciex, Darmstadt, Germany) equipped with in-house packed C18 columns (75 µm inner diameter) of 20 cm length (Reprosil-Pur 120 ODS-3, 3 µm (Dr. Maisch, Ammerbuch, Germany)) without a pre-column. A binary solvent system comprising buffer A (0.5% acetic acid) and buffer B (0.5% acetic acid, 80% acetonitrile) was used for separation. Samples were loaded in 2% buffer B using a flow rate of 500 nl/min. Separation was performed by a gradient from 2% to 35% buffer B in 100 min, followed by a gradient from 35% to 80% in 20 min with a flow rate of 250 nl/min.

Mass spectrometric measurements were performed in the data-dependent mode. The spray voltage was 2.3 kV with no sheath or auxiliary gas flow and the ion transfer tube temperature was set to 200°C. MS spectra in the range of 350 – 2000 m/z were acquired with an automatic gain control (AGC) of 1x10^6^. The resolution was set to 60,000 at m/z 400. The five most abundant multiple charged peptides were fragmented in the linear ion trap using 35% collision energy, with an AGC target of 5000. Dynamic exclusion was enabled with a duration of 90 s.

MS raw files were analysed with MaxQuant (v. 1.5.3.30 (Cox and Mann, 2008)) and the integrated Andromeda search engine (Cox et al., 2011). Generated peak lists were searched against the UniProt protein *mus musculus* sequence database (release 07.11.2013, 51,193 protein entries) and the contamination file supplied with MaxQuant. MaxQuant was operated using default settings with slight modifications. Database search was performed with trypsin as proteolytic enzyme allowing up to two missed cleavages. Oxidation of methionine and N-terminal acetylation were set as variable modifications. Raw data were recalibrated using the “first search” option of Andromeda using a precursor mass tolerance of 20 ppm and a fragment mass tolerance of 0.5 Da. Mass spectra were analysed with Andromeda using default settings. The mass tolerance for precursor and fragment ions was 4.5 ppm and 0.5 Da, respectively. For automated quantification of protein groups, only “razor and unique” peptides and a minimum ratio count of two were considered. In addition, “requantify” and “match between runs” with a 0.7 min matching time window were enabled. A false discovery rate of 1% was applied on both peptide-spectrum-matches and protein lists. Only peptides with a minimum length of seven amino acids were reported. If proteins were not distinguishable based on the set of peptides identified, they were combined by MaxQuant and listed as protein group.

All raw data and original MaxQuant result files have been deposited to the ProteomeXchange Consortium (http://proteomecentral.proteomexchange.org) via the PRIDE partner repository (Vizcaíno et al., 2013) with the data set identifier PXD005072.

Analysis of protein groups was performed with the Perseus software (Tyanova et al., 2016). Contaminants and false positives identified by MaxQuant (see above) were removed from the list. SILAC ratios were swapped, so that all four experiments displayed a Tgfbr2-cKO to WT ratio. Normalized ratios were log2-transformed and a mean log2 was calculated across all 4 replicates. A one-sample t-test was used for statistics. Proteins with a p-value below 0.05 and fold change higher or lower than +1.5 or -1.5 were considered regulated.

**Immunocytochemistry:**

Cells were permeabilized and blocked in 10% horse serum / 0.1% Triton-X100/PBS for 1 hour and primary antibody was applied for over night at 4°C in blocking solution. Cells were washed 3 times in PBS before incubation with fluorophore-coupled secondary antibodies in block solution at room temperature. After 3 times washing with PBS, cells were incubated for 1 min in DAPI solution incubation was followed by 3 more washing steps in PBS. Coverslips were mounted on glass slides with fluorescent mounting medium (#S3023, DAKO, Jena, Germany). The following first and secondary antibodies were used: MFGE8 (goat, 1:100, #AF2805, R&D Systems), GFAP (rat, 1:500, #13-0300, Invitrogen), TUJ1 (mouse, 1:100, MMS-435P, Covance), NG2 (rabbit, 1:200, ab5320, Merck Millipore), HuC/D (mouse, 1:100, A21271, Invitrogen), TBR2 (rabbit, 1:500, ab23345, Abcam), GFP (chicken, 1:1000, ab13970, Abcam), CRE (mouse, 1:500, MAB3120, Millipore), mCherry (rabbit, 1:200, ab167453, Abcam), donkey-anti-goat-Alexa488 (1:500, A-11055, Life-Technologies) or -Cy3 (1:500, 705-165-147, Dianova), donkey-anti-rabbit-Alexa488 (1:500, 711-545-152, Dianova) or -Alexa594 (1:500, 711-585-152, Dianova), donkey-anti-chicken-Alexa488 (1:500, 703-545-155, Dianova), donkey-anti-mouse-Alexa488 (1:500, 715-545-151, Dianova) or -Alexa594 (1:500, 715-585-151), donkey-anti-rat-Alexa488 (1:500, 712-545-153, Dianova) or -Alexa594 (1:500, 712-585-153, Dianova) and donkey-anti-rat-AMCA (1:200, 712-155-153, Dianova).

**MFGE8 ELISA**:

Levels of MFGE8 were quantified in conditional medium (CM) collected from Tgfbr2-cKO DT and VT cell cultures from DIV12. CM was centrifuged for 10 min at 17,000 rcf prior to use to remove particles. The ''mouse MFG-E8 Quantikine ELISA Kit'' (#MFGE80, R&D systems) was used according to manufacturer instructions. Triplicates of each sample and duplicates of the standard (0.125 - 4 µg/ml) were prepared and incubation was performed overnight at 4°C. ELISA signal was quantified with a Multiskan FC Microplate Photometer (Thermo Scientific). Absorbance was measured at 570 nm and corrected to the absorbance at 450 nm. Standard protein curve was fitted with a four parameter logistic before protein amounts were calculated. Graphs and statistics were done with GraphPad Prism.

***In situ* cell death detection (TUNEL):**

ICCs were performed as described above. After incubation with DAPI stain, TdT-mediated dUTP-X nick end labelling (TUNEL) was applied according to the manufacturer instructions (In Situ Cell Death Detection Kit, TMR red, #12156792910, Roche-Diagnostic). Shortly, enzyme and substrate solution were mixed in a ration 1:10 and coverslips with cells were incubated with the solution for 30 min at 37°C. After three subsequent washing steps with PBS coverslips were mounted.

**Immunoelectron microscopy**:

E13.5 Tgfbr2-cKO and control brains were fixed with 4% PFA/0.1 M phosphate buffer with 0.2% or 0.5% glutaraldehyde for 2 hours, embedded in albumin/gelatine and cut into 100 µm. Sections were blocked in 10% horse serum for 8 hours and were incubated over night with MFGE8 antibody (mouse, 1:100, Santa Cruz Biotechnology (SCBT), Heidelberg, Germany) in 10% horse serum/PBS. After washing, sections were incubated in 1.4 nm gold-coupled goat-anti-mouse (1:100) in 2% normal goat serum/tris buffer saline (TBS) over night at 4°C. Sections were postfixed in 1% glutaraldehyde in 25 mM PBS for 10 min. Silver enhancement (HQ-Silver, Nanoprobe, Yaphank, NY, USA) was performed according to manufacturer's instruction for 8 min at 10 - 15°C. After incubation in 0.5% OsO4 in phosphate buffer for 40 min sections were dehydrated with increasing concentrations of ethanol (50%-100%). At 70% ethanol, sections were incubated with 1% uranyl acetate for 35 min. Next, sections were incubated first in propylene oxide/Durcupan mixture, followed by pure Durcupan before they were mounted. 60-70 nm sections were cut with a Leica UC6 (Leica, Wetzlar, Germany) and images were taken with an Electron microscope LEO906E (Zeiss) with the sharp-eye 2k CCD camera (Trödle, Moorenweis, Germany).

**Immunohistochemistry on cryo sections:**

PFA fixed brains were embedded in TissueTec (SAKURA, Torrance, CA, USA) cut in 14 µm sections and mounted on SuperFrost Plus Microscope slides (Thermo Scientific). Prior to staining, sections thaw for 5 min at 37°C and washed in PBS. Permeabilization and blocking was performed for 1 hour in 10% horse serum/0.1% Triton-X100/PBS and primary antibodies were applied for over-night in blocking solution. Sections were washed 3 times in PBS and incubated with secondary antibody in blocking solution for 1 hour. After 3 washing steps in PBS, incubation with DAPI for 5 min and final 3 times wash, sections were cover slipped in fluorescent mounting medium (#S3023, DAKO). The following first and second antibodies were used: MFGE8 (goat, 1:100, #AF2805, R&D Systems), GFAP (rat, 1:500, rat, 1:500, #13-0300, Invitrogen), S100B (rabbit, 1:1000, Abcam), ALDH1L1 (rabbit, 1:500, ab56777, Abcam), NG2 (1:200, ab5320, MerckMillipore), OLIG2 (rabbit, 1:500, #AB9610, MerckMillipore), IBA1 (rabbit, 1:500, weg-2172, Wako), donkey-anti-goat-Alexa488 (1:500, A-11055, Life-Technologies) or -Cy3 (1:500, 705-165-147, Dianova), donkey-anti-rabbit-Alexa488 (1:500, 711-545-152, Dianova) or -Alexa594 (1:500, 711-585-152, Dianova), donkey-anti-rat-Alexa488 (1:500, 712-545-153, Dianova) or -Alexa594 (1:500, 712-585-153, Dianova).

**Immunohistochemistry with thick sections:**

E13.5 forebrains or six week old mouse brains were fixed for 24 hours in 4% PFA and sectioned at 50 µm. Sections were permeabilized and blocked in 10% horse serum/0.2% Tween20/PBS for 3 hours and incubated with primary antibody diluted in blocking solution for 48 hours. Sections were washed 3 times in PBS and secondary antibodies were applied for either 3 hours at room temperature or over-night at 4°C. Sections were washed in PBS, incubated 10 min in DAPI solution, washed again in PBS and mounted with fluorescent mounting medium (#S3023, DAKO). The following first and secondary antibodies were used: GFAP (rabbit, 1:500, Z0334, DAKO), MFGE8 (goat, 1:100, #AF2805, R&D Systems), MFGE8 (mouse, 1:100, sc-30394, SCBT), MFGE8 (rabbit, 1:100, sc-33546, SCBT), PDGFRb (mouse, 1:100, 14-1402-82, eBioscience), Laminin (rabbit, 1:100, ab11575, Abcam), GFP (chicken, 1:1000, ab13970, Abcam), donkey-anti-goat-Cy3 (1:500, 705-165-147, Dianova), donkey-anti-rabbit-Alexa594 (1:500, 711-585-152, Dianova) and donkey-anti-chicken-Alexa488 (1:500, 703-545-155, Dianova).

**Imaging:**

Wide field fluorescence images were obtained with a microscope Imager Z2 (Zeiss). Confocal images were obtained with a laser scanning confocal microscope SP8 (Leica). Images were processed with the Zen and LASX software, respectively. Final figures were prepared using FIJI (ImageJ, v. 2.0.0-rc-43/1.51d (Schindelin et al., 2012)) and Inkscape (v. 0.91).

**Quantification of cell percentage:**

Cells and DAPI positive nuclei were counted with the ''Particle Counter'' macro from FIJI in overlay images. Cells were normalized to DAPI stained nuclei. For the MFGE8 astrocyte quantification (Fig. 4 F-J) MFGE8 opsonized cells were excluded from quantification. Graphs and statistical analyses were done with GraphPad Prism (v. 6.07).

**Reporter plasmid cloning, packaging of lentiviral particles, transduction and induction of astrocyte differentiation:**

The loxP-mCherry-STOP-loxP cassette (source plasmid kindly provided by Dr. Touc, University Medicine Göttingen, (Parthasarathy et al., 2014)) was cloned into a pLenti3-2A-EGFP-no-Puro backbone (Applied Biological Materials Inc., Richmond, BC, Canada) by cutting with EcoR1 and Kpn1 and ligation into the pLenti3-2A-EGFP backbone. The resulting pLenti3-loxP-mCherry-STOP-loxP-2A-GFP was packaged into lentiviral particles according to the protocol described previously (Hellbach et al., 2014; Vezzali et al., 2016). Foxg1^cre/+^;Tgfbr2^flox/+^ and WT DT and VT cells were transduced 4 hours after seeding with 4 infecting units/cell of lentiviral particles. At DIV6 astrocyte differentiation was induced by changing the medium to DMEM / 10% FCS / 1% PSN / 1% glutamine. Cells were fixed at DIV12 for 15 min with 4% PFA / PBS. Immunostainings were performed as described above without DAPI staining.

pLenti-NeuroD1-Cre-NLS_RFP-2A-Puro, Ad:GFAP-Cre (Merkle et al., 2007), pLenti-CMV-Cre-NLS_RFP-2A-Puro, and pLenti-CMV-RFP-2A-Puro were used to transduce E13.5 Tgfbr2^flox/flox^ DT and VT cells. Transduced cells were selected with puromycin (0.3 μg/ml), starting 6 days before harvesting at DIV12, and cell extracts for immunoblotting were prepared as outlined above.

**Flow cytometry:**

Brains were dissected and cerebral cortex, caudate putamen and hippocampus were isolated. All three regions were separately collected in cold buffer (1x HBSS (Hanks’ balanced salt solution) containing 1 mM EDTA and 1% BSA. Tissue was subsequently homogenized using a glass homogeniser followed by filtering through 75 µm cell strainers. Samples were centrifuged 12 min at 300xg and 10°C. Dissociated cells were resuspended in 0.9 M sucrose prepared in HBSS and centrifuged for 10 min at 700xg. The supernatant containing the myelin was removed, and the pelleted cells were washed with HBSS. Finally, cells were transferred to PBS containing 1% FCS and centrifuged for 5 min, 200xg, 4°C. Cells were stained with primary and secondary antibodies using the following concentrations: MFGE8 (goat, 1:100, #AF2805, R&D Systems), Zenon® Alexa Fluor® 647 Goat IgG Labelling Kit, GFP (chicken, 1:1000, ab13970, Abcam), donkey-anti-chicken-Alexa488 (1:500, 703-545-155, Dianova), GFAP (rabbit, 1:500, Z0334, DAKO), PE Donkey anti-rabbit IgG (1:1000, #406421, Biolegend). Cells were analysed using the BD Accuri C6 flow cytometer (BD, Heidelberg, Germany). Gates for detecting positive staining were set against unstained and only secondary antibody- stained controls. Data were analysed using BD™ CFlow® Plus software.

Supplementary References:

Cox, J., and Mann, M. (2008). MaxQuant enables high peptide identification rates, individualized p.p.b.-range mass accuracies and proteome-wide protein quantification. *Nat. Biotechnol.* 26, 1367–1372. doi:10.1038/nbt.1511.

Cox, J., Neuhauser, N., Michalski, A., Scheltema, R. A., Olsen, J. V., and Mann, M. (2011). Andromeda: A Peptide Search Engine Integrated into the MaxQuant Environment. *J. Proteome Res.* 10, 1794–1805. doi:10.1021/pr101065j.

Hellbach, N., Weise, S. C., Vezzali, R., Wahane, S. D., Heidrich, S., Roidl, D., et al. (2014). Neural deletion of Tgfbr2 impairs angiogenesis through an altered secretome. *Hum. Mol. Genet.* 23, 6177–6190. doi:10.1093/hmg/ddu338.

Merkle, F. T., Mirzadeh, Z., and Alvarez-Buylla, A. (2007). Mosaic Organization of Neural Stem Cells in the Adult Brain. *Science* 317, 381–384. doi:10.1126/science.1144914.

Parthasarathy, S., Srivatsa, S., Nityanandam, A., and Tarabykin, V. (2014). Ntf3 acts downstream of Sip1 in cortical postmitotic neurons to control progenitor cell fate through feedback signaling. *Development* 141, 3324–3330. doi:10.1242/dev.114173.

Rappsilber, J., Mann, M., and Ishihama, Y. (2007). Protocol for micro-purification, enrichment, pre-fractionation and storage of peptides for proteomics using StageTips. *Nat. Protoc.* 2, 1896–1906. doi:10.1038/nprot.2007.261.

Schindelin, J., Arganda-Carreras, I., Frise, E., Kaynig, V., Longair, M., Pietzsch, T., et al. (2012). Fiji: an open-source platform for biological-image analysis. *Nat. Methods* 9, 676–682. doi:10.1038/nmeth.2019.

Tyanova, S., Temu, T., Sinitcyn, P., Carlson, A., Hein, M. Y., Geiger, T., et al. (2016). The Perseus computational platform for comprehensive analysis of (prote)omics data. *Nat. Methods* advance online publication. doi:10.1038/nmeth.3901.

Vezzali, R., Weise, S. C., Hellbach, N., Machado, V., Heidrich, S., Vogel, T., et al. (2016). The FOXG1/FOXO/SMAD network balances proliferation and differentiation of cortical progenitors and activates Kcnh3 expression in mature neurons. *Oncotarget* 5. Available at: http://www.impactjournals.com/oncotarget/index.php?journal=oncotarget&page=article&op=view&path%5B%5D=9545 [Accessed May 28, 2016].

Vizcaíno, J. A., Côté, R. G., Csordas, A., Dianes, J. A., Fabregat, A., Foster, J. M., et al. (2013). The Proteomics Identifications (PRIDE) database and associated tools: status in 2013. *Nucleic Acids Res.* 41, D1063–D1069. doi:10.1093/nar/gks1262.

Supplementary Legends

**Supplementary Figure S1: Heavy amino acid incorporation in E13.5 forebrain cells**

(A) Schematic overview: E13.5 forebrain SILAC cultures and experimental layout. Histograms of peptides, which contained either Lys4 or Arg6 (B) or Lys8 or Arg10 (C) compared to their unlabeled counterparts at DIV6 (left panels) and DIV12 (right panels). Both labels are only partially incorporated into the proteome at DIV6, but labelling is virtually complete at DIV12.

**Supplementary Figure S2: MFGE8 localizes primarily in the proximity of blood vessels in the telencephalon at E13.5**

(A) MFGE8 immunofluorescence is located mainly near blood vessels in the E13.5 telencephalon from WT and from Tgfbr2-cKO animals. In the Tgfbr2-cKO high immunofluorescence is also observed at previously described endothelial cell clusters. Scale bar: 200 µm, n=3. (B) IHCs of WT and Tgfbr2-cKO telencephalon with MFGE8 and IB4, PDGFRb or Laminin. MFGE8 signal surrounds IB4 positive EC, but localizes inside PDGFRb positive pericytes and co-localizes with LAMININ in the basal lamina of blood vessels in WT and Tgfbr2-cKO telencephalon. n=3, Scale bar: 25 μm. (C) Immunolabeled electron microscopy of WT and Tgfbr2-cKO vessel in the telencephalon. MFGE8 localizes in the basal lamina surrounding the ECs. n=2, Scale bar: 1 μm. (D) Representative fluorescence images of DT and VT cultures after immunofluorescence for neuronal marker HuC/D (n=4), intermediate progenitor marker TBR2 (n=4), NG2 (n=4), TUNEL (n=3) and MFGE8 (n=3). (E) The graph shows the percentage of each one of these cell types in cultures. Mean with SEM. Scale bar: 100 µm.

**Supplementary Figure S3: MFGE8 and GFAP distribution in the neonatal (P0) and juvenile brain (P21), and MFGE8 localisation in adult brain, where it does not co-localize with microglia or oligodendrocytes**

(A) Immunofluorescence with antibodies against MFGE8 and GFAP of P0 and P21 brains (a + c: caudal and b + d: rostral sections). Scale bar: 200 µm. From each section and time point magnifications of cortex (CTX), dentate gyrus (DG) and caudate putamen (cPA) are shown. Scale bar: 50 µm. (B) Immunofluorescence with antibodies against MFGE8 and S100B or ALDH1L1 of adult brains. Magnifications of cortex (CTX), hippocampus (HIP) and caudate putamen (cPA) are shown. Magenta arrowheads indicate S100B+ or ALDHL1L+ cells, white arrowheads indicate MFGE8+ cells and yellow arrowheads indicate double positive cells (MFGE8+/S100B+ or MFGE8+/ALDHL1+). Scale bar: 50 µm. (C) Immunofluorescence with antibodies against MFGE8 and microglia marker IBA1 shows no co-localisation in cortex (CTX) or dentate gyrus (DG). MFGE8 does not co-localize with the oligodendrocyte markers OLIG2 or NG2, respectively. MFGE8-positive cells labeled with white arrowheads, IBA1+, OLIG2+ and NG2+ labeled with magenta arrowheads. Scale bar: 100 µm, n=1.

**Supplementary Figure S4:**

(A) ELISA analysis of conditioned media (CM) from DIV12 DT and VT cells of Tgfbr2-cKO and respective controls shows reduced MFGE8 secretion in CM derived from DT of the Tgfbr2-cKO, but not from VT. Mean with SEM; **:p<0.01; unpaired Student's t-test, n=8. (B) Quantification of TUNEL+ cells in DT and VT cultures from wild type (WT) cells (n=3) and (C) Tgfr2b-cKO cells (n=3). (D) Quantification of TUNEL+/MFGE8+ cells in DT and VT cultures from wild type (WT) cells (n=3) and (E) Tgfr2b-cKO cells (n=3). Mean with SEM; unpaired Student's t-test

**Supplementary Figure S5: FOXG1-expressing progenitor cells give rise to GFAP+ astrocytes *in vitro***

(A) Illustration of the reporter plasmid. CRE recombinase removes the mCherry-STOP cassette and thereby enables GFP expression. (B) Immunostainings of GFAP, mCherry and GFP in FOXG1-cre expressing and WT (control) cells from E13.5 DT and VT after transduction with the reporter plasmid and subsequent astrocyte differentiation. GFAP and GFP co-localisation (yellow arrowhead) is identified in DT and VT FOXG1-cre expressing cells. GFAP and mCherry co-expression (magenta arrowheads) is observed in DT and VT cells from WT forebrains. Scale bar: 40 µm, n=3.

**Supplementary Figure S6: GO-term analysis of genes expressed specifically in MFGE8 or GFAP-positive astrocytes reveals evidence for functional divergence of these subtypes.**

Gene ontology analysis of (A) MFGE8 and (B) GFAP astrocyte population according to Cahoy et al., 2008 MFGE8 astrocytes affect for example endothelial cells, whereas GFAP astrocyte might influence neuronal differentiation.

**Supplementary Figure S7: Original immunoblots of key findings in Fig. 4**

(A-E) Original Immunoblots used for representation in Fig. 4 A-E. Relevant bands are marked with a yellow box.

**Supplementary Figure S8: Original immunoblots of key findings in Fig. 5**

(A-D) Original Immunoblots used for representation in Fig. 5 A-D. Relevant bands are marked with a yellow box.
