# Supplementary figures and images for: TGFβ-Signaling and FOXG1-Expression Are a Hallmark of Astrocyte Lineage Diversity in the Murine Ventral and Dorsal Forebrain
Source: Front Cell Neurosci. 2018 Nov 28;12:448. doi: 10.3389/fncel.2018.00448 (PMC6282056; doi:10.3389/fncel.2018.00448)

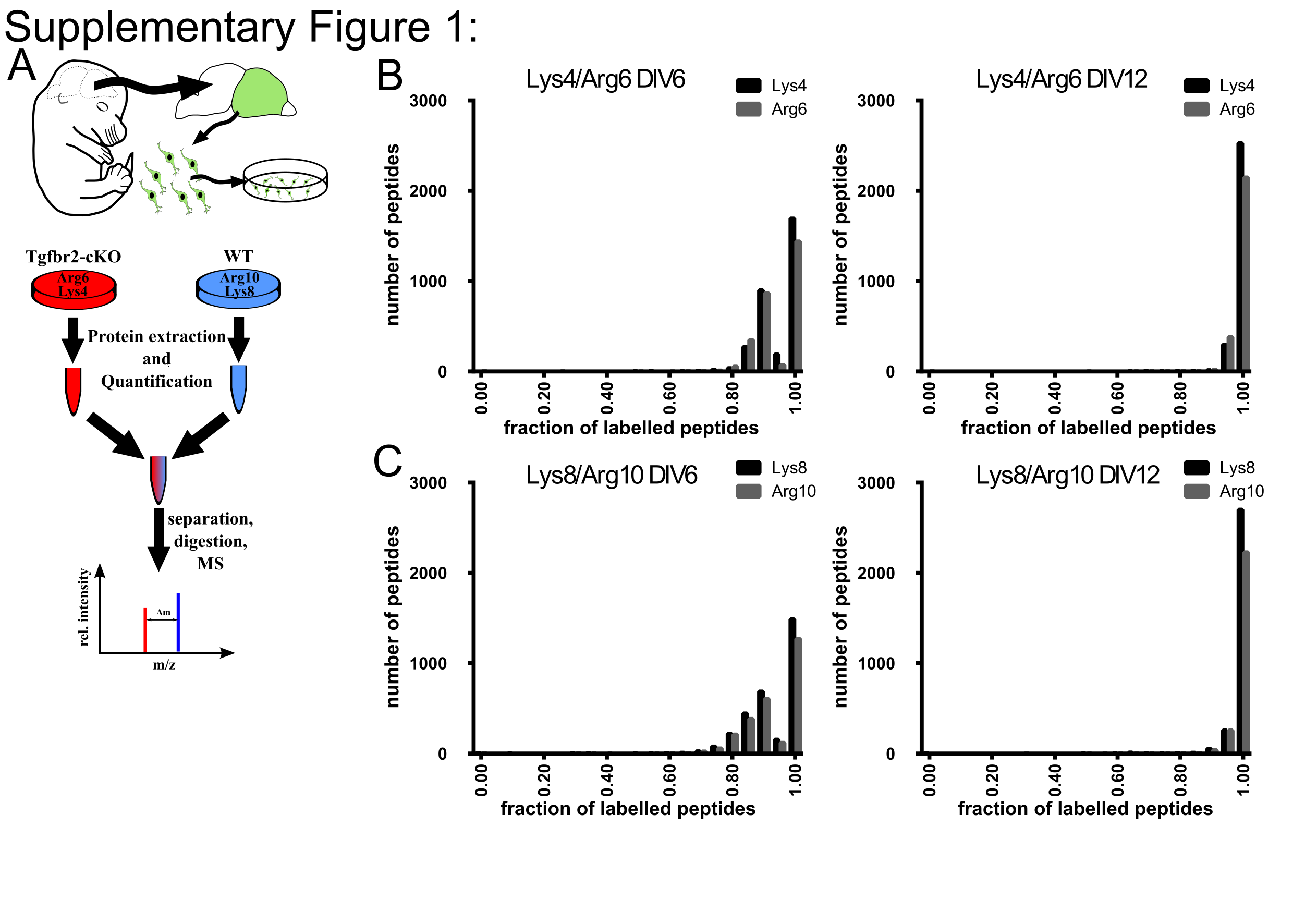

Supplement: Supplementary file 2 [file Image_1.TIF]

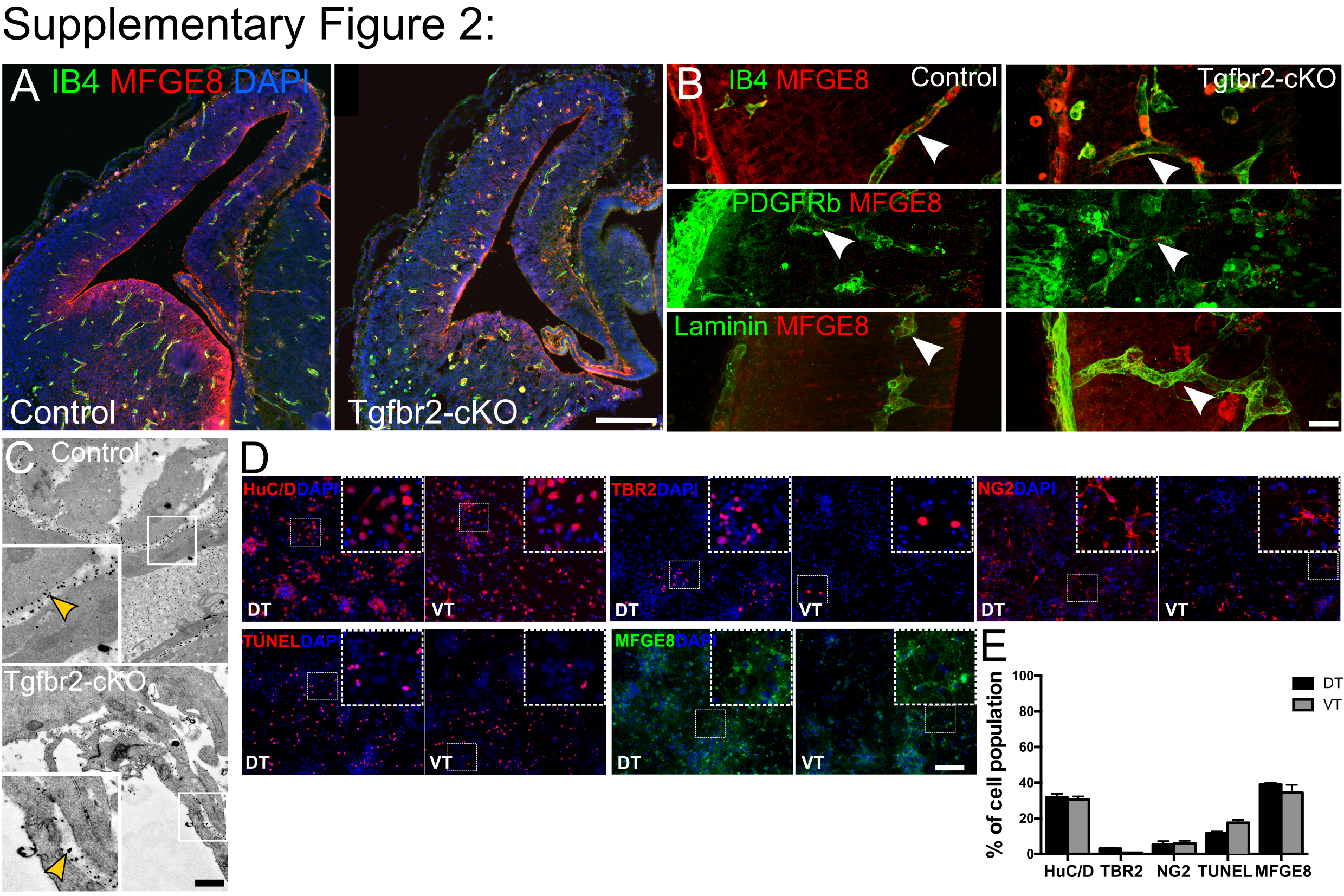

Supplement: Supplementary file 3 [file Image_2.TIF]

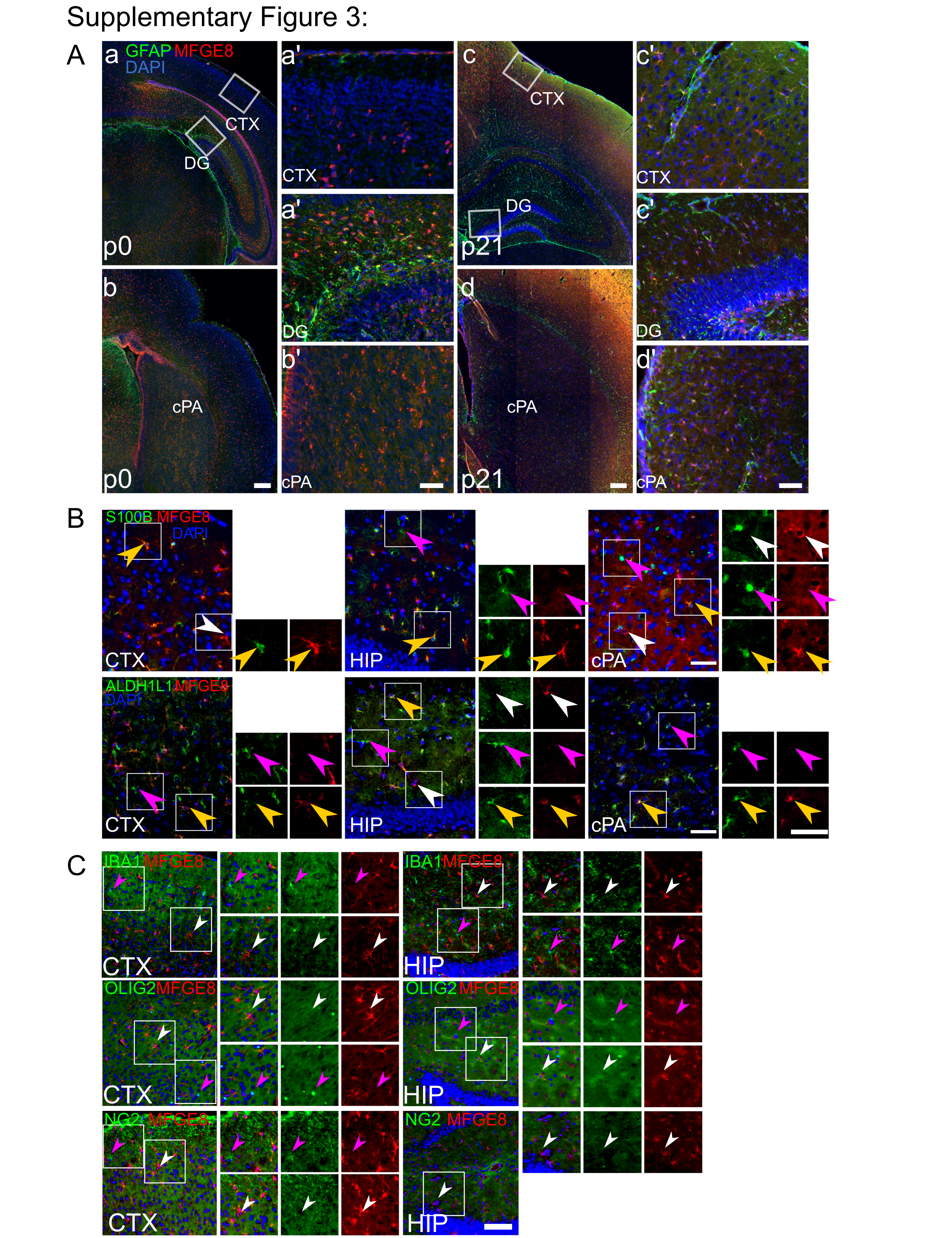

Supplement: Supplementary file 4 [file Image_3.TIF]

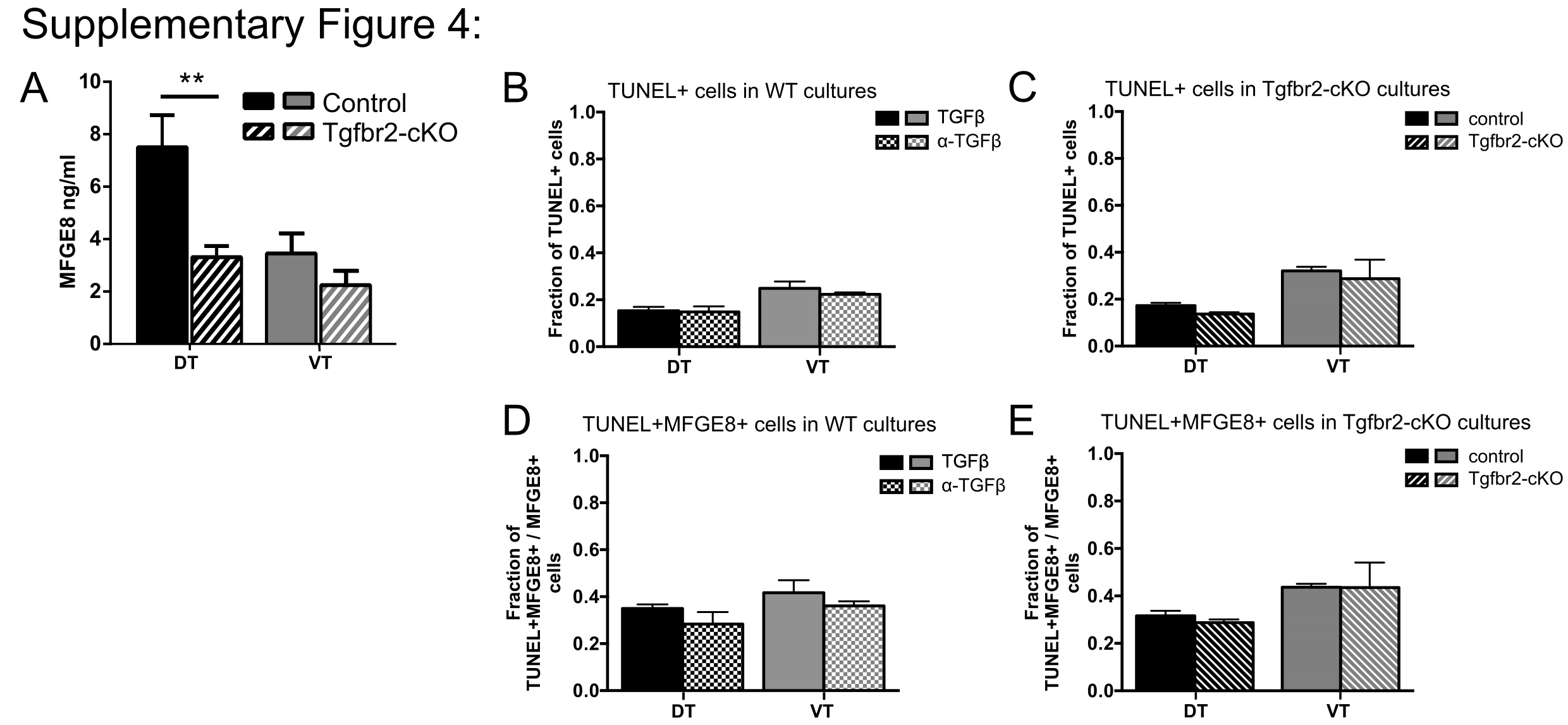

Supplement: Supplementary file 5 [file Image_4.TIF]

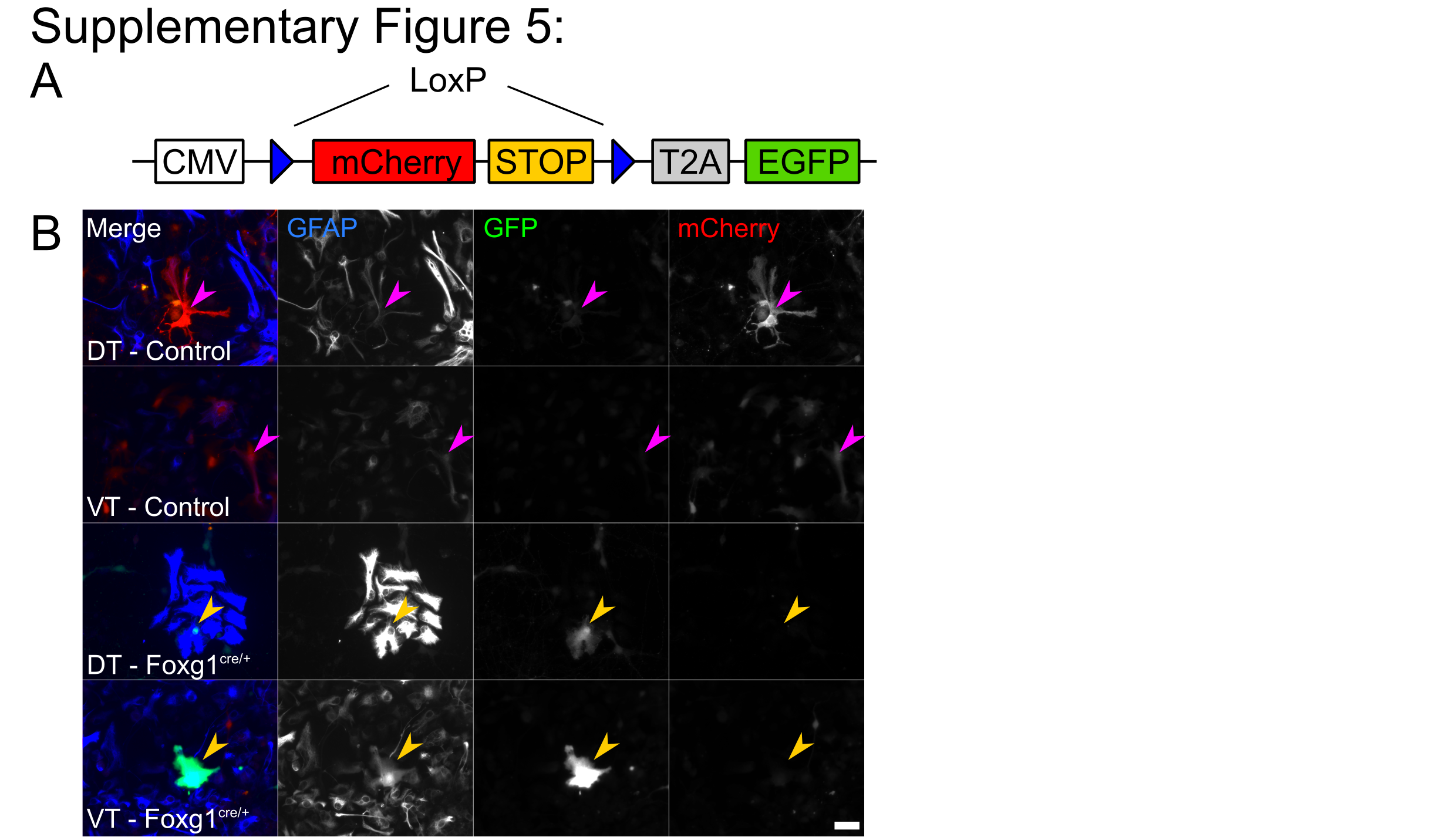

Supplement: Supplementary file 6 [file Image_5.TIF]

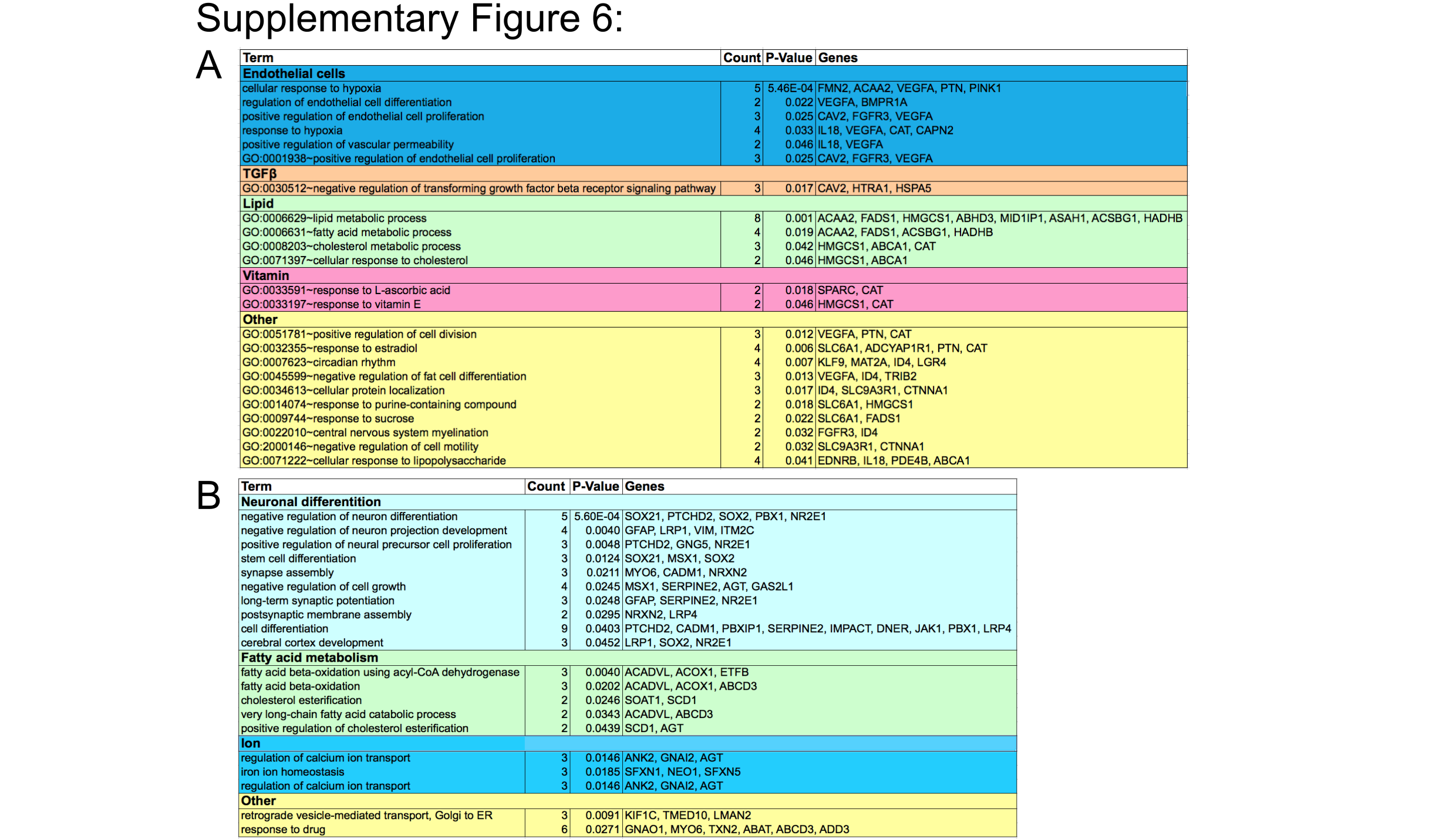

Supplement: Supplementary file 7 [file Image_6.TIF]

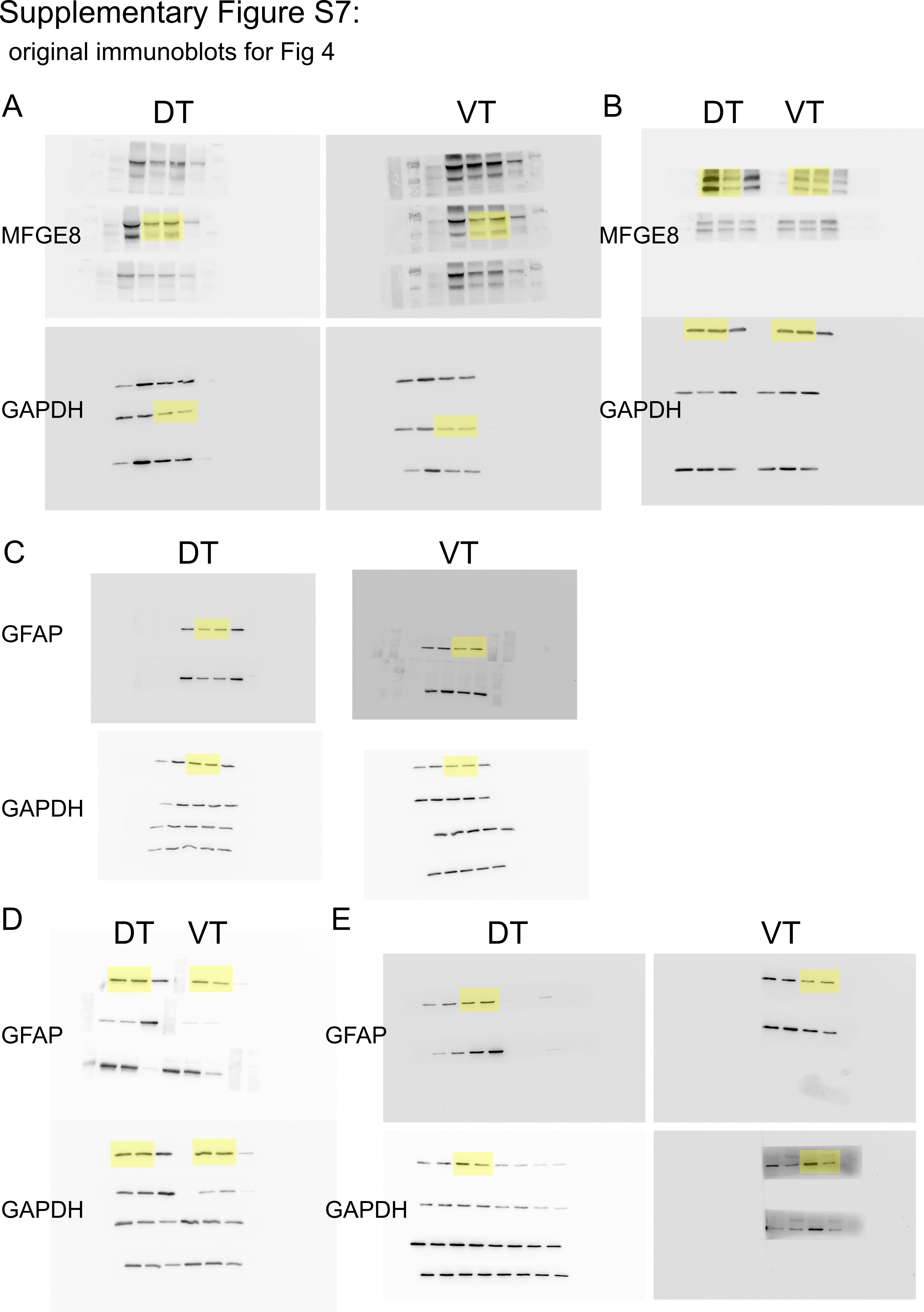

Supplement: Supplementary file 8 [file Image_7.tif]

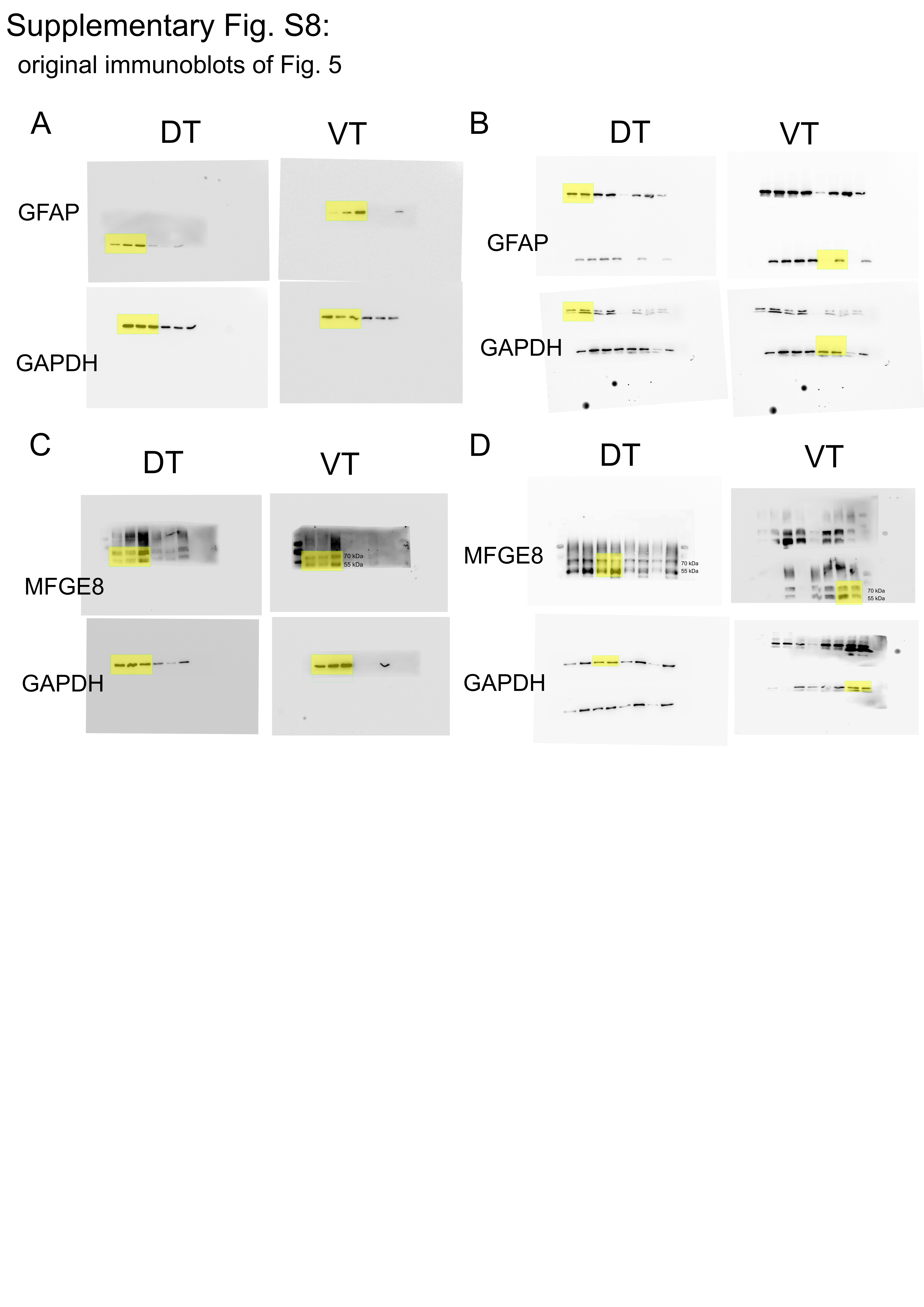

Supplement: Supplementary file 9 [file Image_8.tif]
